# An innovative intramedullary bone graft harvesting concept as a fundamental component of scaffold-guided bone regeneration: A preclinical *in vivo* validation

**DOI:** 10.1016/j.jot.2024.05.002

**Published:** 2024-06-05

**Authors:** Markus Laubach, Buddhi Herath, Sinduja Suresh, Siamak Saifzadeh, Bronwin L. Dargaville, Silvia Cometta, Victoria Schemenz, Marie-Luise Wille, Jacqui McGovern, Dietmar W. Hutmacher, Flavia Medeiros Savi, Nathalie Bock

**Affiliations:** a Australian Research Council (ARC) Training Centre for Multiscale 3D Imaging, Modelling, and Manufacturing (M3D Innovation), Queensland University of Technology, Brisbane, QLD 4000, Australia; b Centre for Biomedical Technologies, School of Mechanical, Medical and Process Engineering, Queensland University of Technology, Brisbane, QLD 4000, Australia; c Department of Orthopaedics and Trauma Surgery, Musculoskeletal University Center Munich (MUM), LMU University Hospital, LMU Munich, Munich, Germany; d Jamieson Trauma Institute, Metro North Hospital and Health Service, Royal Brisbane and Women's Hospital, Herston, QLD 4029, Australia; e Max Planck Queensland Centre (MPQC) for the Materials Science of Extracellular Matrices, Queensland University of Technology, Brisbane, QLD 4000, Australia; f Abteilung für Zahnerhaltung und Präventivzahnmedizin CharitéCentrum 3 für Zahn-, Mund- und Kieferheilkunde Charité – Universitätsmedizin Berlin, Berlin, Germany; g ARC Training Centre for Cell and Tissue Engineering Technologies, Queensland University of Technology, Brisbane, QLD 4000, Australia; h Medical Engineering Research Facility, Queensland University of Technology, Chermside, QLD 4032, Australia; i Translational Research Institute, Woolloongabba, QLD 4102, Australia; j School of Biomedical Sciences, Faculty of Health, Brisbane, Queensland University of Technology, Brisbane, QLD 4000, Australia

**Keywords:** Bone graft, Bone regeneration, Intramedullary harvesting, Polycaprolactone, Scaffold, Voronoi

## Abstract

**Background:**

The deployment of bone grafts (BGs) is critical to the success of scaffold-guided bone regeneration (SGBR) of large bone defects. It is thus critical to provide harvesting devices that maximize osteogenic capacity of the autograft while also minimizing graft damage during collection. As an alternative to the Reamer-Irrigator-Aspirator 2 (RIA 2) system – the gold standard for large-volume graft harvesting used in orthopaedic clinics today – a novel intramedullary BG harvesting concept has been preclinically introduced and referred to as the ARA (aspirator + reaming-aspiration) concept. The ARA concept uses aspiration of the intramedullary content, followed by medullary reaming-aspiration of the endosteal bone. This concept allows greater customization of BG harvesting conditions vis-à-vis the RIA 2 system. Following its successful *in vitro* validation, we hypothesized that an ARA concept-collected BG would have comparable *in vivo* osteogenic capacity compared to the RIA 2 system-collected BG.

**Methods:**

We used 3D-printed, medical-grade polycaprolactone-hydroxyapatite (mPCL-HA, wt 96 %:4 %) scaffolds with a Voronoi design, loaded with or without different sheep-harvested BGs and tested them in an ectopic bone formation rat model for up to 8 weeks.

**Results:**

Active bone regeneration was observed throughout the scaffold-BG constructs, particularly on the surface of the bone chips with endochondral bone formation, and highly vascularized tissue formed within the fully interconnected pore architecture. There were no differences between the BGs derived from the RIA 2 system and the ARA concept in new bone volume formation and in compression tests (Young's modulus, *p* = 0.74; yield strength, *p* = 0.50). These results highlight that the osteogenic capacities of the mPCL-HA Voronoi scaffold loaded with BGs from the ARA concept and the RIA 2 system are equivalent.

**Conclusion:**

In conclusion, the ARA concept offers a promising alternative to the RIA 2 system for harvesting BGs to be clinically integrated into SGBR strategies.

**The translational potential of this article:**

Our results show that biodegradable composite scaffolds loaded with BGs from the novel intramedullary harvesting concept and the RIA 2 system have equivalent osteogenic capacity. Thus, the innovative, highly intuitive intramedullary harvesting concept offers a promising alternative to the RIA 2 system for harvesting bone grafts, which are an important component for the routine translation of SGBR concepts into clinical practice.

## Graphical abstract

**Figure:**
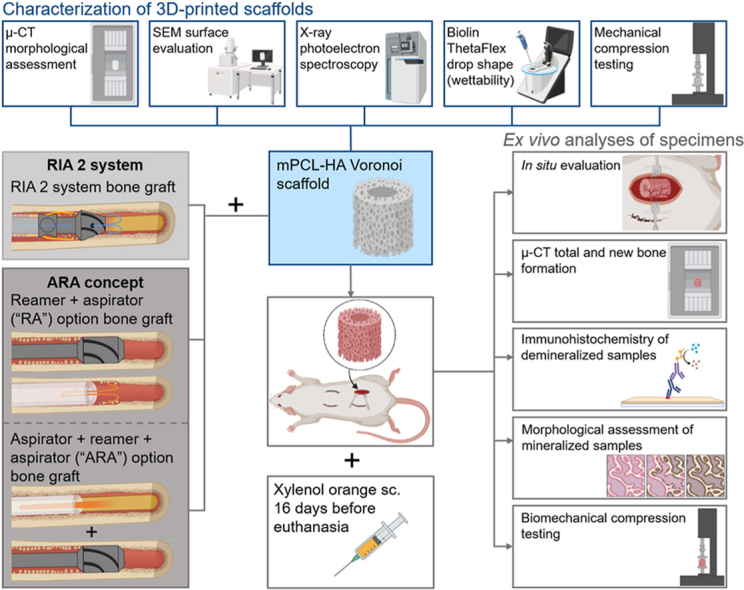

## 1 Introduction

Following a series of successful large animal trials, the concept of rectilinear infilled, three-dimensional (3D)-printed, medical-grade polycaprolactone (mPCL)-ceramic composite scaffolds has attracted the attention of clinicians as a potential alternative treatment for bone defects [1]. Those scaffolds have been applied in clinics as pivotal tools in the concept termed “scaffold-guided bone regeneration” (SGBR) with encouraging results, achieving functional bone tissue regeneration [2,3]. Notably, the recent advancements in additive manufacturing technology platforms have enabled the fabrication of scaffolds made of medical-grade polymers and composites leading to improved morphologies not requiring additional support materials and extensive post-processing [4]. In particular, the Voronoi design of 3D-printed, mPCL-hydroxyapatite (mPCL-HA) scaffold with a complex internal morphology (referred to as Generation 4.0 scaffold, see Suppl. Fig. 1) was found to exhibit suitable and slow *in vitro* degradation behaviour (<1 % mass loss within 6 months) and favourable *in vivo* biocompatibility when combined with bone grafts (BGs), as typically used in clinical SGBR settings [5].

The SGBR concept encompasses an instructive scaffold design strategy in which structural scaffold morphology includes high porosity, full 3D interconnectivity of pores, and appropriate pore size (>150 μm), facilitating cell growth within the scaffolds and integration of the implant into the host tissue [6]. By simultaneously incorporating BG into the macroscopic scaffold pores, additional osteoinductive and osteogenic capacities are introduced, ultimately leading to enhanced bone regeneration [1,7]. Yet, due to its heterogeneous composition made of a variety of cellular and extracellular components and the strenuous collection process, BG quality needs to be thoroughly assessed preclinically within scaffold-BG combinations to warrant future clinical uptake and the success of the SGBR concept. Both shape and mechanical properties of scaffolds used for SGBR need to be maintained during the initial phase of bone regeneration, but their degradation should only start when the remodelling of newly formed bone is initiated [8,9]. Thus, if the biodegradable implant is developed in combination with BG for a function where it must withstand mechanical loading (at least to some extent), as is the case for large defect regeneration, further evaluation of the scaffold-BG combinations *in vivo* is needed [10].

Using cultured cell lines or stem cells from a patient's blood or bone marrow as an alternative to autologous BG transplantation has been proposed to reduce harvest morbidity [11]. However, there are persistent and considerable scientific and regulatory concerns for using cells seeded on scaffolds [12] by Food and Drug Administration (FDA) and ce (“Conformité Européenne”, French for “European conformity”) marks, which are currently being intensively debated, requiring, for instance, comprehensive and expensive Phase III clinical trials for cell therapies in combination with biomaterials [[13], [14], [15]]. Furthermore, previous studies have shown that without additional extracellular matrix (ECM) as is present in fresh BG, the cells alone are not able to achieve sufficient bone regeneration in combination with scaffolds [12,16,17]. Hence, for the success of the SGBR concept, it is crucial to utilize fresh BG, which contains an ECM with a reservoir of complete and specific structural and signalling proteins at a physiological dose and in a nonrecombinant state [18,19].

In the clinic, for the reconstruction of large bone defects within the context of SGBR, a BG is usually harvested from the intramedullary canal of long bones using the Reamer-Irrigator-Aspirator (RIA) 2 system (Synthes) [20,21]. A novel harvesting concept, described as the aspirator + reaming-aspiration concept (“ARA” concept), has recently been developed for harvesting of intramedullary BG [5,21]. The ARA concept allows bone marrow to be harvested first, followed by the sequential reaming and iterative use of a new aspirator device to harvest intramedullary BG [21]. The application of this novel harvesting concept enables the collection of different graft material compositions [21]. The two main options for this include (1) the BG of primarily bone chips without bone marrow [termed as the reamer + aspirator (“RA”) option] and (2) a combination of bone marrow and bone chips [termed as the aspirator + reamer + aspirator (“ARA”) option]. Unlike the RIA 2 system, the ARA concept boasts an intuitive design, functions without irrigation during the BG harvest [21], and is further facilitated by the use of standard flexible intramedullary reamers (which are commonly used by surgeons), thus eliminating a steep learning curve [11,22]. Another disadvantage of the RIA 2 system BG is that bone marrow and bone chips are harvested at the same time and therefore the ratio of bone marrow to bone chips in the harvest cannot be controlled [23]. This leads to large variations in graft composition between patients, which is important to consider because relevant proportions of bone chips in BGs are necessary to achieve sufficient osteogenic capacity for bone defect regeneration [5,21], especially as the osteogenic capacity of bone marrow is strongly dependent on age, body fat content, and disease state [[24], [25], [26]].

In our previous preclinical study using the ARA concept, the capacity to preserve the osteoimmune microenvironment of BG associated with high osteogenic potential was demonstrated *in vitro* and proven similar to BG collected with the RIA 2 system [21]. However, this successful *in vitro* study requires *in vivo* preclinical confirmation before moving to large animal studies and clinical trials [27,28]. Hence, we determined in this study the potential of the new ARA concept by evaluating the *in vivo* osteogenic capacity of the BG harvest, loaded in a 3D-printed, Voronoi scaffold made of mPCL-HA, in a rat model of ectopic bone formation (EBF) [5]. Considering the use of the scaffold biomaterials represents an elementary part of the SGBR concept, hence we first undertook these characterizations. Then, we assessed *in vivo* biocompatibility, osteogenic potential, and biomechanical profile of the scaffold-BG constructs, comparing BG collected with the RIA 2 system with BG collected with the novel ARA harvesting concept.

## 2 Materials and methods

### 2.1 Scaffold design and manufacturing

The scaffolds were designed in-house using the software suite Rhinoceros 3D & Grasshopper (Robert McNeel & Associates, Seattle, USA) in accordance with the Voronoi tessellation [29], in which a uniform region can be divided into discrete cells and whose design is based on a random set of points called “seed points” [30]. The Voronoi lattice structure was derived by tessellating a uniform tubular cylinder with an outer diameter of 10 mm, inner diameter 4 mm, and a height of 12 mm, with 70 seed points and a strut diameter of 1 mm. The final Voronoi scaffold design was exported in binary stereolithography (STL) file format (Suppl. Fig. 2) and shared with the manufacturer. Scaffolds were additively manufactured using a composite material comprising mPCL with 4 % HA (Evonik Industries AG, Essen, Germany) under ISO 13485 certification by BellaSeno GmbH (Leipzig, Germany).

### 2.2 Characterization of 3D-printed scaffolds

#### 2.2.1 Evaluation of morphology and porosity

All micro-computed tomography (μCT) scanning was performed using a Scanco Medical AG μCT 50 scanner (Scanco, Brüttisellen, Switzerland) at a maximum electric potential of 55 kVp and a current of 145 μA. The manufacturing quality was evaluated by assessing the fabrication accuracy in terms of the distribution of HA particles and strut diameters by scanning the scaffolds (n = 2) at high resolution in air with an isotropic voxel size of 1.2 μm^3^ (integration time 800 ms, 0.5 mm aluminium filter). Surface morphology of non-implanted (control) scaffolds was studied by scanning electron microscopy (SEM) in which the scaffold surfaces were platinum sputtered, and images were obtained using a TESCAN MIRA 3 instrument (Tescan, Brno, Czech Republic) operating at an accelerating voltage of 5.0 kV, beam intensity of 8.0, and at a working distance of 8.0 mm. Surface chemical characterization of mPCL-HA scaffolds (n = 6) was performed using X-ray photoelectron spectroscopy (XPS) (AXIS Ultra, Kratos Analytical, UK). Survey spectra were recorded at 6 random locations on each scaffold at a pass energy of 150 eV. Atomic concentrations of the elements present on the surface were calculated from the survey spectra using the CasaXPS software. Further surface characterization included wettability measurements using the Biolin ThetaFlex drop shape (Biolin Scientific, Sweden). Briefly, a water droplet (2 μL) was applied to flat scaffolds, and the contact angle was imaged and quantified using the OneAttention software.

Control scaffolds allocated for evaluation of morphology and porosity were wrapped in low-density foam to prevent movement and scanned in air with an isotropic voxel size of 14.8 μm^3^ followed by conducting 3D reconstructions to obtain the as-manufactured geometry (referred to as μCT models) using Amira 2020.2 (ThermoFischer Scientific, USA). The reconstructed μCT model for scaffolds was imported into the software Rhinoceros 3D & Grasshopper to determine scaffold porosity [5]. The porosity of a structure refers to the percentage of the internal pore volume in its total volume (Suppl. Fig. 3). In addition, open-source image processing software ImageJ (National Institutes of Health, USA) was used to calculate the distribution of pore sizes and the strut diameters of the control scaffolds using μCT images of the scaffolds (Suppl. Fig. 4).

For mechanical testing, the control scaffolds were immersed in a 1*X* phosphate buffered saline (PBS; pH 7.4; Sigma–Aldrich) bath under physiological conditions (37 °C) for 15 min to achieve testing temperature, and unconfined tests under continued simulated physiological conditions were performed with a strain rate of 0.1 mm/s using a 500 N load cell on an Instron model 5567 instrument (Melbourne, Australia). The set-up for the mechanical testing of the mPCL-HA Voronoi scaffolds is shown in Supplementary Fig. 5. We used the calculated slope of the initial linear region of the fitted stress–strain curve as the elastic (Young's) modulus and yield strength was measured at 0.2 % offset strain.

### 2.3 *In vivo* experiments

Ethical approval was obtained from the Queensland University of Technology (QUT) Animal Ethics Committee (UAEC) (Ethics Approval Numbers 2000000592 and 2,000,000,593). The animal surgeries were performed at the QUT Medical Engineering Research Facility (MERF), the Prince Charles Hospital campus (Chermside, Queensland, Australia). The study was conducted in accordance with the requirements of the Australian Code of Practice for the Care and Use of Animals for Scientific Purposes, and the ARRIVE 2.0 guidelines (Animal Research: Reporting of *In Vivo* Experiments) [31].

#### 2.3.1 Perioperative procedures

##### 2.3.1.1 Harvesting BGs from sheep

**Figure 1:**
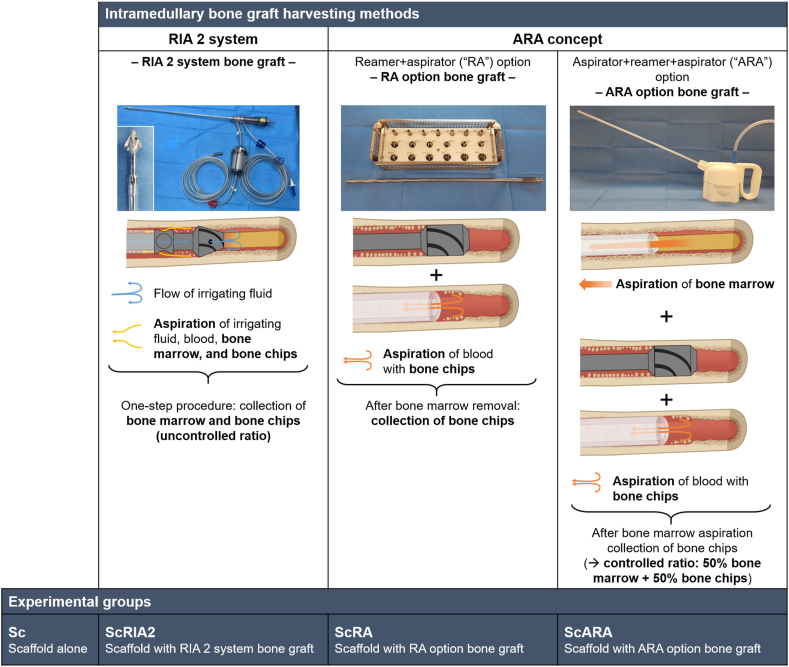
Clinically relevant intramedullary harvesting methods for obtaining BGs from the sheep femur and illustration of the study design in terms of the experimental group composition. Please note the RA option was applied following the removal of bone marrow. The mPCL-HA Voronoi scaffolds alone as well as the mPCL-HA Voronoi scaffolds loaded with BGs obtained from the intramedullary canal of sheep femora were additionally loaded with 120 μL fibrin glue. ARA concept, aspirator + reaming-aspiration concept; RIA 2 system, Reamer-Irrigator-Aspirator 2 system; Sc, scaffold. Adapted from Ref. [21]. Partially created with BioRender.com.

Different types of fresh ovine femoral BGs were harvested from female Merino sheep under sterile conditions. The details of the ovine femoral intramedullary harvesting methods used for BGs and associated experimental groups are listed in Fig. 1 and are described throughout elsewhere [21]. Briefly, from fully anesthetized sheep (n = 8, mean body weight [BW] 44.0 ± [standard deviation, SD] 2.4 kg, 1–2 years of age) BG was harvested using either the RIA 2 system (n = 4) or the ARA concept (n = 4) with the RA or ARA options in order to obtain the RIA 2 system BG, the RA option BG or the ARA option BG. The sheep were humanely killed on the surgery day using 100 mg/kg of pentobarbital sodium (Lethabarb®) intravenously.

##### 2.3.1.2 Construct implantation rats

A total of 10 rats were included to perform conclusive qualitative histological and quantitative biomechanical analyses with a sufficient number of samples. Thus, 10 male CBH-rnu/Arc (nude) rats (aged 12–14 weeks) with mean BW of 208.70 ± 23.04 g were purchased from the Animal Resources Centre (Canning Vale, Western Australia) and housed in individually ventilated double-decker cages (Techniplast, GR1800) in a specific pathogen-free and temperature-controlled environment. A failure of thymus formation (dysgenesis of the thymus) due to being homozygous for the Foxn1^nu^ mutation in these CBH-rnu/Arc (nude) rats leaves no place for CD4^+^ and CD8^+^ T cells to differentiate and mature, making them T cell-deficient [32]. Before starting the experiments, the rats were allowed to acclimatize for at least 1 week with *ad libitum* access to sterile food and water. The rat EBF model recently utilized by our group was applied, including scaffold preparation and perioperative procedures conducted in biosafety cabinets under sterile conditions [5].

The rats received constructs of scaffold-only as well as scaffolds loaded with different types of freshly harvested ovine femoral BGs (1.0 g per scaffold). Fibrin glue (120 μL, TISSEEL Fibrin Sealant, Baxter Healthcare International) was added to the scaffold-only group as well as the different types of BGs loaded onto the scaffolds, which were previously sterilized using gamma irradiation with a dose of 27.7 kGy (Steritech Pty Ltd, Narangba, Australia). Surgical placement of the scaffolds in the rats has been described in detail elsewhere [5]. Briefly, general anaesthesia was induced and maintained with isoflurane inhalation (3–4% for induction and 1–2% for maintenance) in oxygen. Subcutaneous buprenorphine (0.05 mg/kg BW) and meloxicam (1 mg/kg BW) were administered for pre-emptive analgesia. Prophylactic antibiotic (cefazolin 20 mg/kg BW) was given subcutaneously once preoperatively and for 2 days postoperatively. In order to achieve a random distribution of specimens and to implant only 1 construct from each experimental group per rat, a total of 3–4 constructs were implanted into the subcutaneous pockets of each rat (1 construct per pocket). Under sterile conditions, over the rats’ dorsum 1 cm lateral to the spinous processes 3 to 4 skin incisions of ca. 1.3 cm were made forming in total 3 to 4 subcutaneous pockets by blunt dissection below the panniculus carnosus (1–2 pockets per side relative to spinous processes with 1.5 cm in between the incisions). Surgical wounds were closed in layers. Tramadol in drinking water (25 mg/L) was provided for 5 days after surgery for postoperative analgesia.

#### 2.3.2 Fluorochrome dye labelling (during follow-up) and euthanasia after 8 weeks

Labelling with an intravital marker substance was performed for observation of active bone regeneration dynamics. *In vivo* bone labelling using fluorochromes, which bind to bone minerals by chelating calcium ions on the surface of newly formed apatite crystals, has become a standard technique in skeletal research [33]. Thus, the aqueous solution of the fluorochrome dye xylenol orange (Sigma–Aldrich, cat# 398,187) was prepared as per the protocol described elsewhere [34] and administered subcutaneously with a dose of 45 mg/kg BW 16 days before euthanasia. The rats were euthanized by CO_2_ asphyxiation 8 weeks post-implantation.

### 2.4 Ex vivo analyses of specimens

#### 2.4.1 In situ macroscopic assessment and total bone volume analysis using μCT imaging data

After euthanasia, all specimens were macroscopically examined *in situ* for ingrowth into surrounding tissue, blood vessel ingrowth, and signs of inflammation. Further, all specimens underwent μ10.13039/100004811CT scanning in either 70 % ethanol or 1*X* 10.13039/100015823PBS with an isotropic voxel size of 17.2 μm^3^ (integration time 800 ms, 0.5 mm aluminium filter), and the total bone volume was calculated applying a threshold of 270/1000 (which complies with 403.5–1950.0 mg 10.13039/100016376HA/cm³), a 10.13039/100014230Gaussian filter width of 0.8, and gauss filter support of 1 using SCANCO's proprietary scan evaluation software. On average 2 samples from each experimental group were used for a highly specialized representative qualitative histological evaluation including multiple correlative characterizations. Further, to enable robust statistical evaluation, we used ≥5 samples per experimental group for quantitative biomechanical evaluation. Thus, scaffold specimens retrieved from 2 out of the 10 rats (Sc group: 1 sample; ScRIA2 group: 2 samples; ScRA group: 2 samples; ScARA group: 2 samples) were fixed in 4 % paraformaldehyde for 5 days before transferring to 70 % ethanol (v/v) until needed for further qualitative histological analyses. In addition, specimens retrieved from 8 out of the 10 rats (Sc group: 7 samples; ScRIA2 group: 6 samples; ScRA group: 5 samples; ScARA group: 8 samples) were kept unfixed and transferred to 1*X* PBS at pH 7.4 immediately after retrieval for quantitative biomechanical testing.

#### 2.4.2 Paraffin (histology and immunohistochemistry) and resin embedding (morphological assessment)

Fixed specimens were cut longitudinally in two parts using an EXAKT 310 Diamond Band Saw (EXAKT Apparatebau GmbH & Co. KG, Norderstedt, Germany) for subsequent histological analysis of decalcified paraffin-embedded samples as well as undecalcified samples embedded in resin.

##### 2.4.2.1 Histology and immunohistochemistry of demineralized samples

For histological and immunohistochemical (IHC) analyses, samples were decalcified for 17 days, processed, embedded, sectioned and stained according to protocols previously established by our research group [5,35,36]. Supplementary Table 1 lists primary antibodies and protocol specifications.

For staining with tartrate-resistant acid phosphatase (TRAP), the samples were deparaffinized in 2 changes of xylene for 3 min, 2 changes of 100 % ethanol for 2 min, and with 95 %, 70 %, and 50 % ethanol changes for 1 min, respectively. Next, the samples were incubated with 0.1 M acetate buffer (In 50 mL distilled water, 0.41 g of 0.1 M sodium acetate was mixed with 0.58 g of 50 mM L (+)tartaric acid) for 20 min at room temperature, and pH was adjusted to 5.0. Next, samples were incubated with naphthol AS-MX phosphate and fast red TR salt for 2 h (For a 50 mL of 0.1 sodium acetate, 25 mg of 0.5 mg/mL naphthol AS-MX phosphate was mixed with 55 mg of 1.1 mg/mL fast red TR salt). The samples with TRAP staining were then rinsed in distilled water, followed by counterstaining in Mayer's hematoxylin for 2 min. A water-soluble mounting media (Fluoromount-G, Thermo Fisher) was used for the coverslip slides. All stained slides were scanned at 20× objective with a spatial resolution of 0.27 μm using a 3DHistech Scan II Brightfield slide scanner (3DHistech, Budapest, Hungary).

#### 2.4.3 Morphological assessment of mineralized samples

To characterize the differences in the ultrastructure and composition of scaffold–BG constructs – aiming to assess bone regeneration and collagen orientation – undecalcified samples were embedded in resin [35]. As described in detail previously [5,33,37], 5 different assessment techniques were employed: (1) SEM with resin cast etching to visualize the surface morphological features of the BGs and osteocyte network; (2) confocal laser scanning microscopy (CLSM) of xylenol orange to qualitatively confirm bone regeneration; (3) histological analysis using modified Goldner's trichrome (GT) staining; (4) rhodamine staining for in-depth analysis of osteocytes lacuno-canalicular network (LCN), and (5) CLSM for orientation of collagen fibres was assessed using second-harmonic generation (SHG) signal (details of these analyses can be found in Suppl. Table 2).

#### 2.4.4 New bone formation and biomechanical testing

New bone formation within entire samples were calculated using μ10.13039/100004811CT imaging data with determining volume of newly formed bone applying a threshold range of 270/310 (which complies with 403.5–488.2 mg 10.13039/100016376HA/cm^3^), a 10.13039/100014230Gaussian filter width of 0.8, and gauss filter support of 1 using SCANCO's proprietary scan evaluation software. According to previously established protocols [16,17], this threshold range of 270/310 was determined by visual evaluation of 20 random tomograms per sample with 4 samples per group, selecting a range of lower (270) and upper (310) thresholds that best reflected the morphology of the newly mineralized tissue and excluded the original bone chips, as well as the scaffold and soft tissues [38]. Furthermore, the unfixed samples were kept in sterile 1*X* PBS (pH 7.4) and subjected to biomechanical testing within 48 h of specimen retrieval. Uniaxial compression testing under simulated physiological conditions using the same protocol as described above for control scaffolds was performed on the specimens to assess the Young's modulus and yield strength (at 0.2 % offset strain).

### 2.5 Statistical analysis

Statistical analyses were performed using *R* programming software (version 4.0.2; R Foundation for Statistical Computing, Vienna, Austria) with RStudio version 1.3.1073 (RStudio Inc., Boston, MA). A significance level of *p* < 0.05 was chosen. Data are presented as the mean with SD (±). *R* package “rstatix” was used for one-way analysis of variance (ANOVA) to compare the statistical differences between the experimental groups as well as for post hoc testing. For the post hoc test, Tukey's adjustment method for multiple comparisons was applied.

## 3 Results

### 3.1 Characterization of 3D-printed, mPCL-HA voronoi scaffolds

A detailed inspection of the additively manufactured, mPCL-HA scaffolds with Voronoi design revealed macroscopically a large-pore 3D architecture with well-fused filaments (Suppl. Figs. 6A and 6B). Supplementary Video 1 depicts a (reconstructed) μCT scan of an mPCL-HA Voronoi control scaffold. Scaffold porosity was measured as 50.44 ± 3.53 %. A bright edge at the periphery of the mPCL-HA composite scaffold filaments indicates a more decentralized distribution of the HA powder particles in the sub-micrometre range at the edges of the struts (Suppl. Fig. 6C). Scanning electron microscopy of the non-implanted control scaffolds showed a high printing accuracy with corresponding appropriate integrity of the filaments and scaffold surface (Suppl. Fig. 6D). It was observed that a majority of the struts had diameters between 700 μm and 1250 μm with pore diameters showing a GAUSS distribution evenly spread between 100 μm and 2000 μm (Suppl. Figs. 6E and 6F). Mechanical testing revealed a Young's modulus of 50.02 ± 2.83 MPa and yield strength of 3.02 ± 0.16 MPa. Supplementary Video 2 shows a typical compression test of the control scaffold. Furthermore, characterization of the surface properties of mPCL-HA Voronoi scaffolds (n = 6) showed the presence of elemental carbon (83.1 ± 2.2 %), oxygen (16.7 ± 2.2 %), and calcium (0.1 ± 0.2 %) on the surface, as well as a water contact angle of 113.4 ± 7.3° (Suppl. Fig. 7).

### 3.2 In situ macroscopic assessment and total bone volume analysis using μCT imaging data

**Figure 2:**
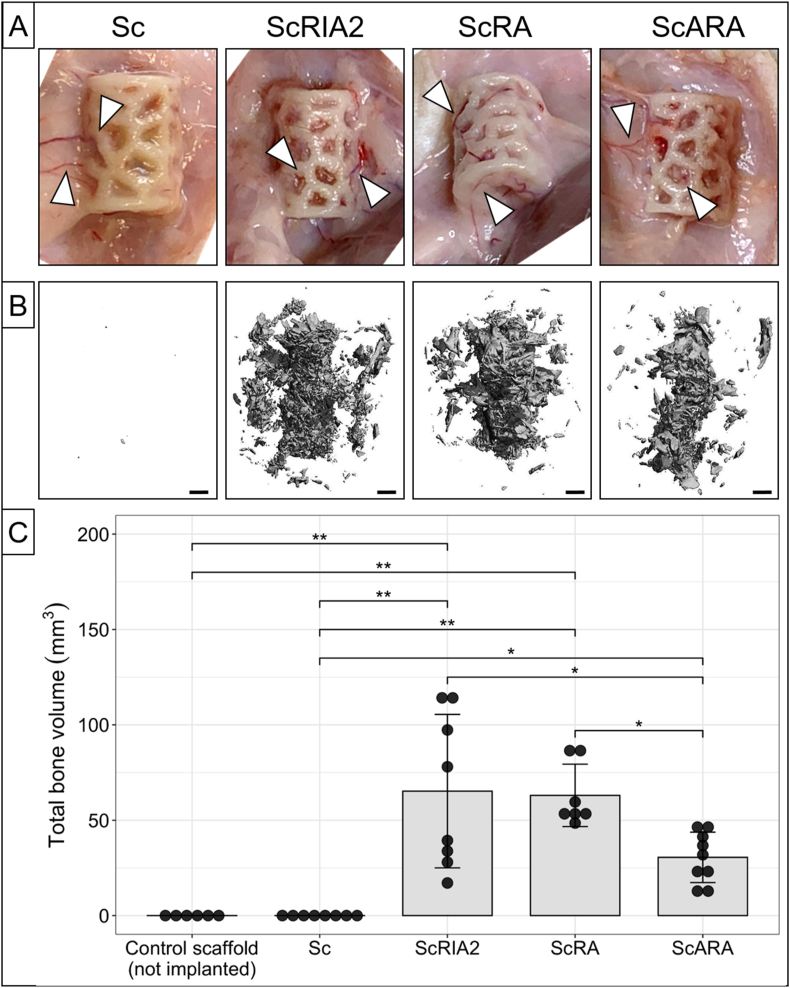
Macroscopic assessments of *in situ* specimens and μCT quantification of total bone volume. During specimen retrieval, excellent tissue integration in all the experimental *in vivo* groups and particularly pronounced ingrowth of blood vessels from surrounding tissue (white triangles) was evidenced (A). In the groups loaded with different types of BG (ScRIA2, ScRA, and ScARA), representative images of reconstructed μCT data show homogenous distribution of the BG throughout the Voronoi scaffolds (B). Please note that the samples of the ScRIA2 and ScRA groups show similar total bone volume; in the ScARA group, about half of the total bone volume is observed compared to the ScRIA2 and ScRA groups (C). Scale bars: B, 1000 μm. Post hoc test Tukey method corrected *p*-values: * <0.05, ** <0.001.

The experimental *in vivo* groups, based on different harvesting methods and associated BGs, assessed hereafter, are shown in Fig. 1 and consisted of the scaffold alone without BG (Sc group) and 3 test groups consisting of scaffold + BG from the RIA 2 system (ScRIA2 group) as well as two ARA concept derived groups: scaffold + BG from the RA option (ScRA group) and scaffold + BG from the ARA option (ScARA group). An overview of the number of samples per experimental group analysed by the different analytical methods can be found in Supplementary Table 3. All rats survived the surgery and the recovery phase and reached the endpoint of the protocol after 8 weeks without adverse events. Following euthanasia, surgical sites and specimens were assessed *in situ* (Fig. 2A). No inflammation or excessive fibrosis was observed around the implanted scaffolds. There was no evidence of 'coalescence' of the specimens and adequate integration with the surrounding tissue was observed, including macroscopically visible ingrowth of blood vessels, in all the experimental *in vivo* groups. Distribution of the BG throughout the scaffolds, including the inner tubular canal and within the pores, was observed in μCT imaging (Sc group: Suppl. Video 3; ScRIA2 group: Suppl. Video 4; ScRA group: Suppl. Video 5; ScARA group: Suppl. Video 6). Furthermore, the quantification of the total bone volume using μCT imaging (Fig. 2B and C) showed, as expected, negligible measured bone volume in the Sc group samples (0.002 ± 0.002 mm^3^). The ScRIA2 group had a total average bone volume of 65.24 ± 40.25 mm^3^ and the ScRA group had a total average bone volume of 63.02 ± 16.38 mm^3^. The ScARA group samples showed total bone volume of 30.53 ± 13.26 mm^3^. The ANOVA statistical analysis indicated overall significant differences between groups (*p* < 0.001) (Fig. 2C). Please note that no significant difference in the post hoc test between ScRIA2 and ScRA groups was observed (*p* = 1) with the largest SD for total bone volume across all groups observed in the ScRIA2 group.

### 3.3 Immunohistochemistry of demineralized samples

**Figure 3:**
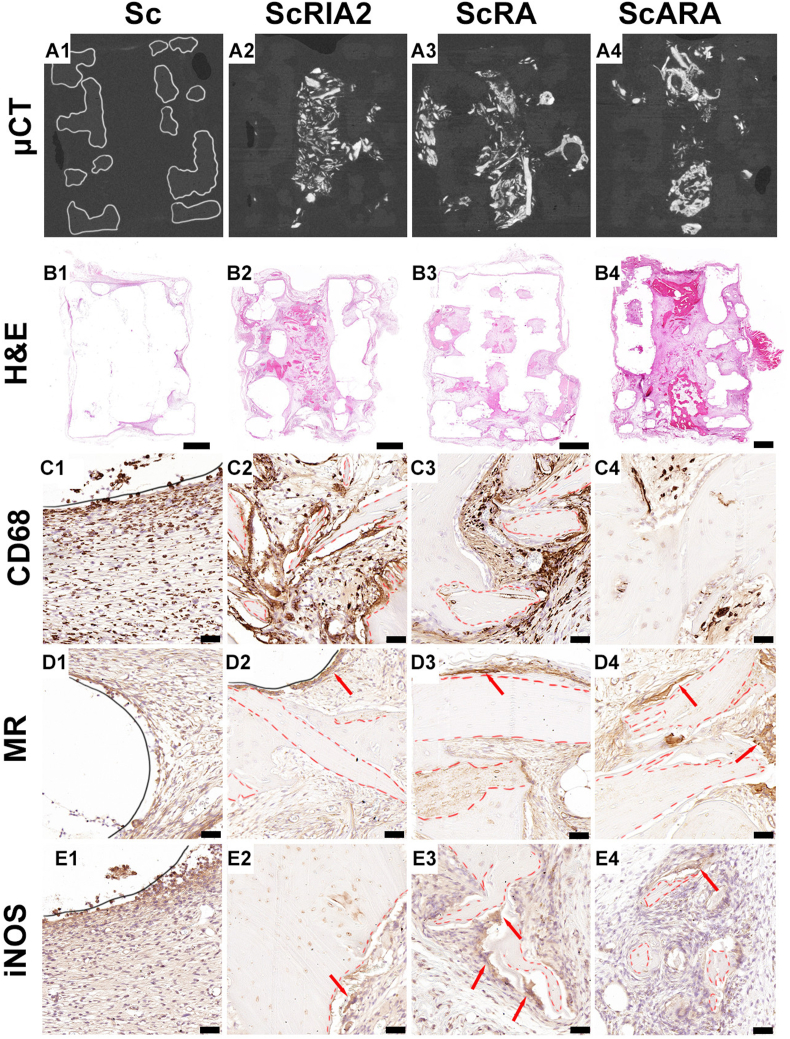
Micro-computed tomography and histological and IHC analysis (inflammatory markers) of explanted mPCL-HA Voronoi scaffolds alone and loaded with different types of BG. Three-dimensional reconstruction of the μCT data of the sample groups after specimen collection (A1–A4). H&E overview (B1–B4) and IHC inflammatory markers (C1–E4). Dashed lines indicate original bone chips. CD68, Cluster of differentiation 68; H&E, hematoxylin and eosin; iNOS, inducible nitric oxide synthase; μCT, micro-computed tomography; MR, mannose receptor. Scale bars: B1–B4, 2000 μm; C1–E4, 50 μm.

**Figure 4:**
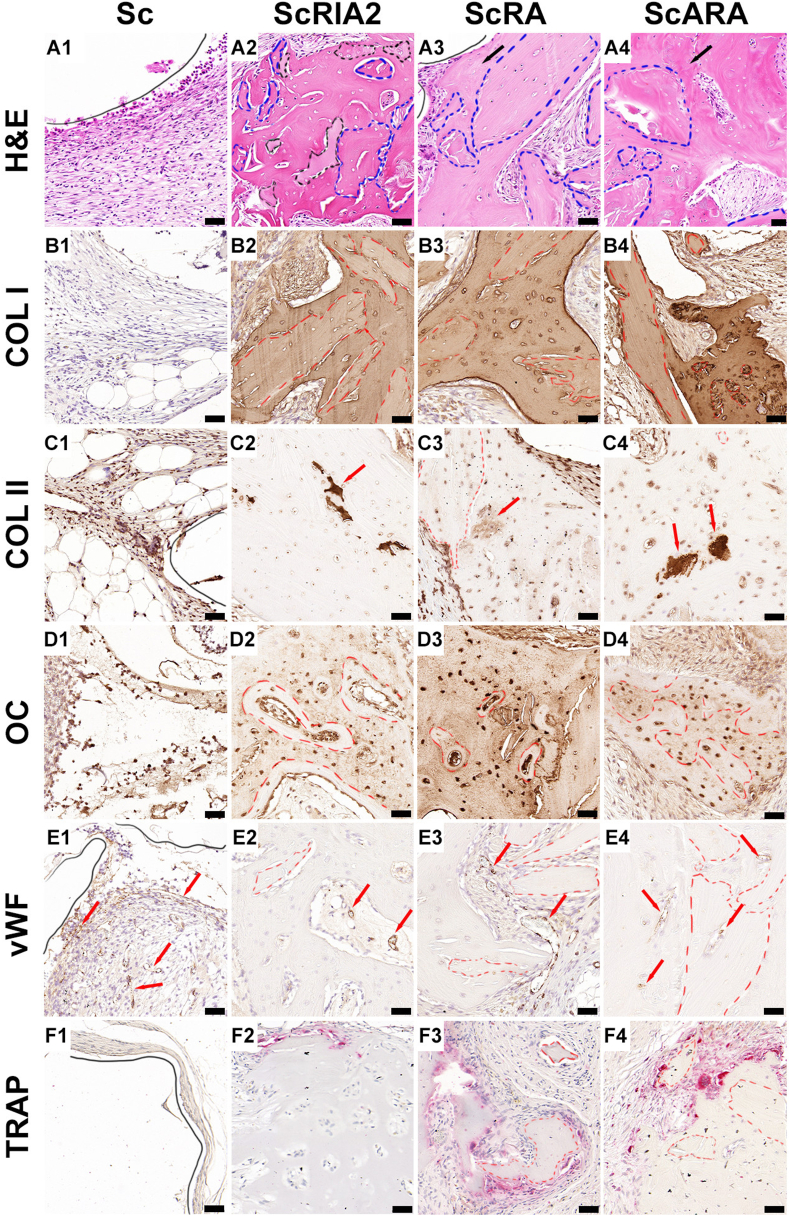
Histological and IHC analysis of explanted specimens. H&E high magnification (A1–A4) and IHC bone formation, vascularization and osteoclastic markers (B1–F4). Dashed lines indicate original bone chips. COL I, collagen type I; COL II, collagen type II; H&E, hematoxylin and eosin; OC, osteocalcin; TRAP, tartrate-resistant acid phosphatase; vWF, von Willebrand factor. Scale bars: 50 μm.

Using IHC performed on the demineralized samples, we focused first on assessing the key regeneration processes; the inflammatory responses, as depicted in Fig. 3; and bone formation and vascularization, as shown in Fig. 4. Using μCT images oriented to the section plane of the IHC samples of the respective groups (Fig. 3A1–3A4) as orientation references, H&E images showed good integration of the scaffolds into the surrounding host tissue in all the *in vivo* groups (Fig. 3B1–3B4). The cellular responses around the mPCL-HA scaffold and around the bone chips were similar across all the *in vivo* groups (Fig. 3C1–3E4). The innate responses to the mPCL-HA scaffold and the various BGs were investigated using CD68 staining, which is particularly expressed by macrophages. Strong staining was observed on the surface of bone chip fragments and in areas of resorbing cartilage (Fig. 3C1–3C4, BG: red dashed lines in C2 and C3). Mannose receptor (MR) reactivity, indicating the release of anti-inflammatory cytokines, was more pronounced on a single layer of cells lining the surface of scaffold struts (Fig. 3D1 & 3D2, black lines), on osteoclasts lining the surface of bone chip fragments (Fig. 3D2–3D4, red arrows), and also on the remnants of the original bone chip fragments (Fig. 3D2–3D4, red dashed lines). Yet, there were a few giant cells that exhibited weak reactive staining for inducible nitric oxide synthase (iNOS), particularly on the cells lining the outer surface of the scaffold struts and bone chip fragments (Fig. 3E2–3E4, red arrows).

Sc group (no BG) showed sparse collagen fibre formation aligning perpendicularly to the outer surface of the scaffold wall on H&E images (Fig. 4A1, black line indicating scaffold strut wall), and this was further confirmed by the lack of collagen type I (COL I) deposition (Fig. 4B1). In all BG groups though (ScRIA2, ScRA, and ScARA), distinguishable stages of mineralization of the extracellular bone matrix could be seen through H&E, including the presence of the original bone chip fragments and small islands of cartilage (Fig. 4A2, blue and black dashed lines, respectively). Areas of bone remodelling could also be identified, including areas with the original bone chips embedded within the woven bone (Fig. 4A3 and 4A4, blue dashed lines and black arrows, respectively). In the same areas, the woven bone was heavily stained for COL I, which is an early osteogenic marker for bone formation and less reactive at the bone chip remnants (Fig. 4B2–4B4, red dashed lines), indicating differences within the new bone matrix but no differences between the experimental groups of scaffolds loaded with different types of BG. The original bone chips, identified by their lamellar morphology and by the presence of islands of hypermineralized cartilage, were positively stained for collagen type II (COL II) (Fig. 4C2–4C4, red arrows), suggesting osteochondral bone formation in all groups of scaffolds loaded with BGs. The bone chips appeared to be alive and stained positively for osteocalcin (OC), a late osteogenic marker of bone formation that was expressed on both osteocytes and bone-lining osteoblasts within the marrow cavity of the forming osteons and woven bone (Fig. 4D2–4D4, red dashed lines). Representative histological sections of the newly forming tissue (exemplified for the ScRA group with H&E, COL I, and OC staining), showing the overlapping and sequential phases of bone remodelling, are shown in Supplementary Fig. 8. The newly formed tissue was well vascularized, as shown by the positive staining of von Willebrand factor (vWF) on the endothelial wall of blood vessels within the soft tissue of Sc group (Fig. 4E1, red arrows) and within the marrow cavities of the remodelling bone tissue of all scaffold-BG groups (Fig. 4E2–4E4, red arrows). Osteoclastic activity, visualized by TRAP staining (Fig. 4F2–4F4), underpinned the osteogenic cellular responses around the mPCL-HA scaffold strut and around the bone chips (Fig. 4F2–4F4, red dashed lines), indicating active bone regeneration throughout the experimental groups of scaffold-BG constructs, although this was absent in the Sc group (Fig. 4F1).

### 3.4 Morphological assessment of mineralized samples

#### 3.4.1 Assessment of active bone regeneration

**Figure 5:**
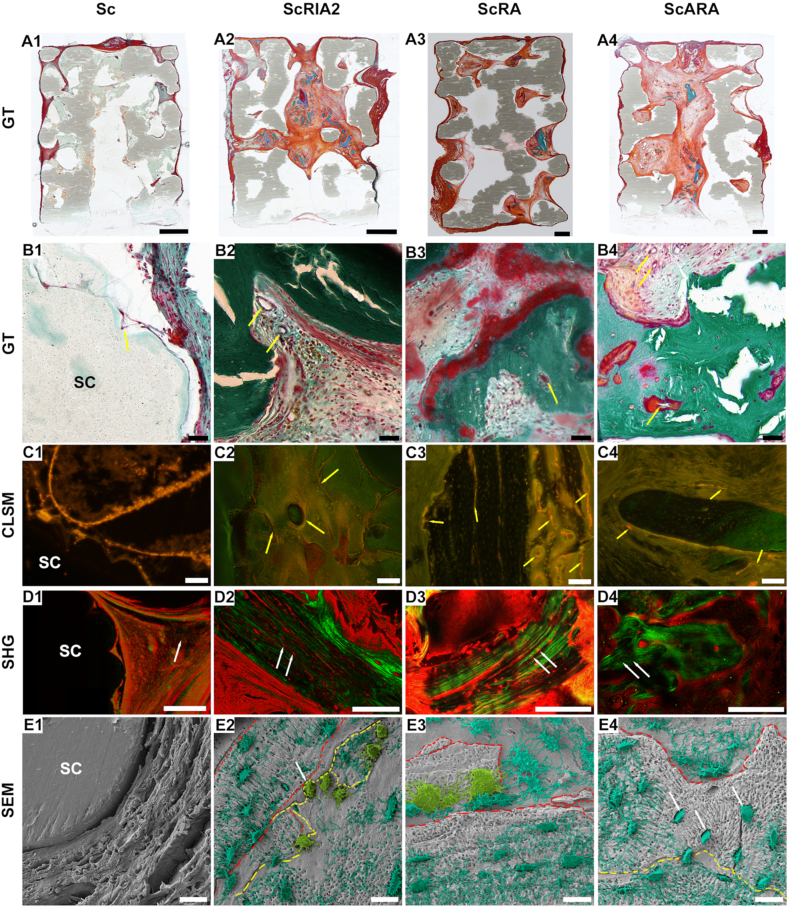
Osteogenesis throughout the scaffold-alone and scaffold-BG constructs assessed with GT (A1–B4), CLSM (C1–C4), SHG (D1–D4) and SEM imaging (E1–E4). Please note the different findings of GT staining: green staining indicates remnants of bone chips, vascularization is indicated by yellow arrows (in B2–B4), red staining indicates osteoid deposits and light blue staining indicates woven bone formation (B1–B4). Dashed lines indicate original bone chips. GT, Goldner's trichrome; CLSM, confocal laser scanning microscopy; SHG, second-harmonic generation; SEM, scanning electron microscopy. Scale bars: A1–A2, 2000 μm; A3–A4, 1000 μm; B1–B4, 50 μm; C1–C4, 100 μm; D1, 200 μm; D2, 200 μm; D3, 250 μm; D4, 250 μm; E1–E4, 20 μm.

Throughout all experimental groups good integration of the scaffold into the surrounding host tissue was observed as demonstrated by the overall morphology of GT staining (Fig. 5A1–5A4). Collagen fibres were aligned around the outer surface of the mPCL-HA scaffold (Fig. 5B1) in the Sc group and around the bone chip fragments of the ScRIA2, ScRA, and ScARA groups (Fig. 5B2–5B4). Although the original architecture of some of the implanted bone chip fragments was still apparent and empty lacunae were observed in some areas, active bone formation could be recognized by the presence of osteoid seams close to areas of hypermineralized cartilage (Fig. 5B3) and at the osteoblasts lining the periosteal surface of the graft fragments and by the formation of small marrow cavities (Fig. 5B3–5B4). The new tissue formed was also well vascularized (Fig. 5B3–5B4, black and yellow arrows). The active and overlapping process of new bone formation was further corroborated by the administration of the xylenol orange subcutaneously 16 days before euthanasia. The graft surfaces were intensely labelled with xylenol orange, fluorescing within the forming bone units, within the remodelling marrow cavity, and within the original graft (Fig. 5C2–5C4, yellow arrows); however, this was not seen in the Sc group (Fig. 5C1).

#### 3.4.2 Assessment of collagen orientation and osteocyte LCN

Excellent scaffold integration into the host tissue in all the *in vivo* groups, especially at the interface between the scaffold struts and the surrounding connective tissue, was observed (Fig. 5D1 and Suppl. Fig. 9). In some parts of the scaffold, the rhodamine and SHG signals were weak (Fig. 5D1 and Suppl. Figs. 9A and 9B); however, the new collagen fibres formed on the surface of the bone chips were arranged radially and parallel to the scaffold struts (Fig. 5D1). Furthermore, rhodamine staining revealed the presence of numerous osteocytes within the mineralized matrix of the lamellar bone as well as the newly formed bone between scaffold struts and bone chips (Fig. 5D2–5D4, Suppl. Figs. 9E and 9F). In areas of woven bone, osteocytes and their LCNs were randomly oriented, the collagen fibres were thinner (Suppl. Figs. 9E and 9F, yellow arrows) as opposed to the lamellar bone, where the osteocytes and their LCNs were organized and collagen fibres appeared to be organized and thicker (Suppl. Fig. 9F, red arrow). The remnants of the original BG of the ScRIA2 and ScRA groups could be clearly identified by their lamellar and ordered structure, with osteocytes axially aligned to collagen fibres, and their LCN perpendicularly aligned to the collagen fibres (Fig. 5D2–5D3, white arrows; Suppl. Figs. 9E and 9F). When present, the collagen fibres of osteons were radially arranged around the Haversian canal (Suppl. Figs. 9E and 9F, white arrows).

The morphology of the osteocytes within the remodelling bone chips, in addition to showing a disturbed canalicular network, appeared to be larger and plump compared to the regions on the surface of the original bone chips, which exhibited an elongated, fusiform body shape (Fig. 5E2–5E4). Corroborating the rhodamine and SHG results, the SEM analysis of the scaffold-alone group (Sc group) showed thick collagen fibres aligned on the outer surface of the scaffold, as well as around the outer scaffold struts and no osteocyte network formation (Fig. 5E1, Suppl. Figs. 10A–10E). In the BG-loaded scaffold groups (ScRIA2, ScRA, and ScARA groups), the remnants of the xenograft material – the lamellar bone – appeared less cellular with the canaliculi running either parallel or perpendicular along the longitudinal axis of flattened osteocytes (Fig. 5E2 and 5E3, dark green osteocytes LCN). A clear demarcation between the regions could be seen at the BG tissue (Fig. 5E2–5E4, red dashed lines). Bone chip resorption preceding bone formation was clearly visible adjacent to these areas (Fig. 5E2 and 5E4, yellow dashed line), with bone resorbing osteoclasts removing a quantum of mineralized bone (Fig. 5E2 and 5E3, light green osteoclasts). This interface seems to be ruffled (Fig. 5E2, yellow dashed line), the tissue appears less porous, and the LCN appears to be broken and partially resorbed (Fig. 5E2–5E4, green canaliculi). Yet, a Howship's lacunae (resorption lacunae) on the surface of the mineralized bone can be clearly seen (Fig. 5E2, white arrow). Moreover, tiny bone chip fragments of the original graft embedded within the new tissue formed were evident, as well as a few apoptotic osteocytes (Fig. 5E3, red dashed line and Fig. 5E4, white arrows, respectively, and Suppl. Figs. 10F, 10G, 10K, 10 P–T). Additionally, areas of woven bone were also present. These areas presented several grouped osteoblast-osteocyte-like cells, with the LCN passing within the bone matrix in irregular and poorly defined arrangement (Suppl. Figs. 10F, 10G, 10K, 10 L, and 10 M, black arrows). These areas also presented remnants of mineralized cartilage (Suppl. Figs. 10G and 10H, blue dashed line and 10I and 10 J) and were highly vascularized (Suppl. Figs. 10F and 10G, 10 K–O, and 10 P, red arrows). In summary, while collagen deposition around the struts was observed in the Sc group, no active bone regeneration was identified in this group. Conversely, in the other *in vivo* groups, active bone regeneration was observed without any qualitative differences between the different scaffold-BG combinations (ScRIA2, ScRA, and ScARA).

### 3.5 New bone formation and biomechanical analysis

**Figure 6:**
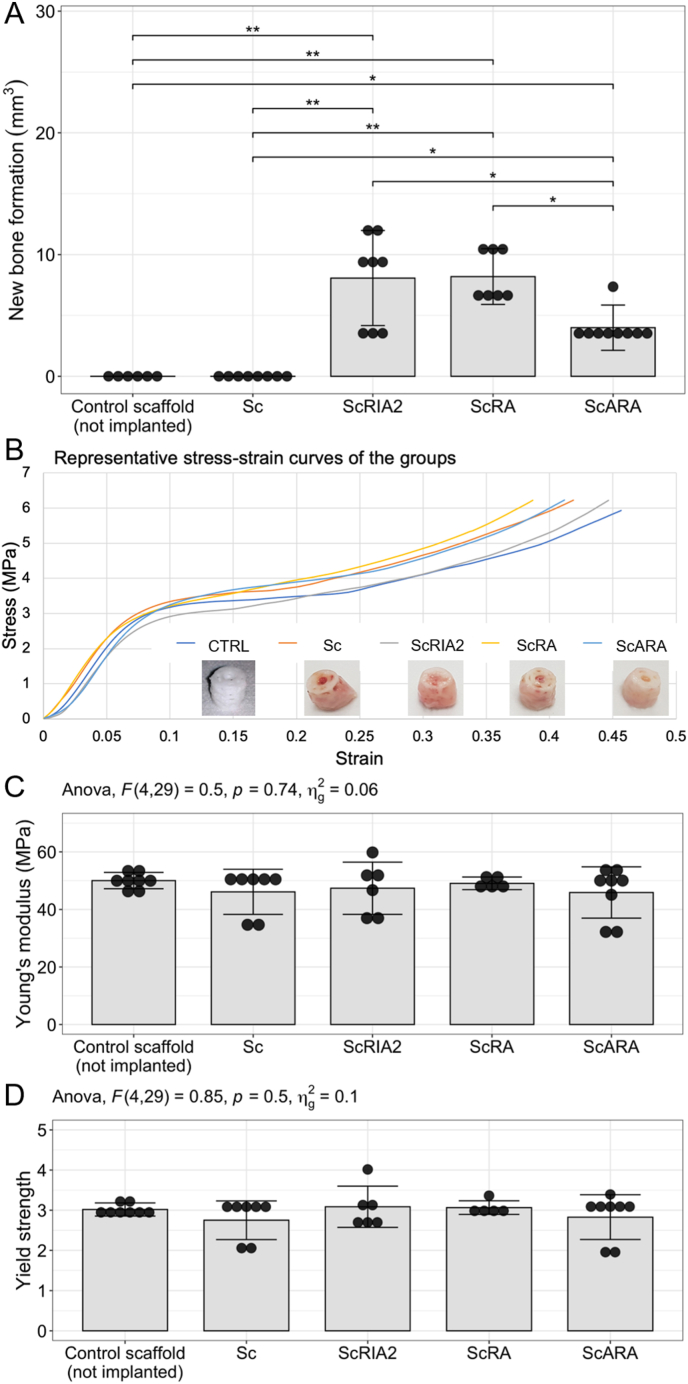
New bone formation and biomechanical analysis. New bone formation quantified from μCT imaging data (A). Exemplary stress–strain curves and sample photos following compression testing (B). Unconfined biomechanical compression testing under simulated physiological conditions showed no significant differences in the one-way ANOVA analysis between the experimental groups for the Young's modulus (C) and yield strength (D). CTRL, control scaffold (not implanted).

Here we assessed the mechanical properties of the scaffold-BG combinations with the hypothesis that implanted scaffolds without BG (Sc group) would have similar properties as the (non-implanted) control scaffold and as the scaffold-BG groups, considering PCL degradation is expected to be minimal within 8 weeks *in vivo*. New bone formation was, as expected, nihil in the Sc group (0.00030 ± 0.00025 mm^3^) compared to the experimental groups of implanted scaffolds loaded with different types of BG (Fig. 6A). Similar new bone formation was observed in the ScRIA2 group (8.07 ± 3.90 mm^3^) and the ScRA group (8.20 ± 2.29 mm^3^, *p* = 1). Approximately 50 % and thereby significantly less new bone formation was observed for ScARA (4.00 ± 1.87 mm^3^) compared to ScRIA2 (*p* = 0.0059) and ScRA (*p* = 0.0063). In summary, the two experimental groups with the largest initial bone chip fraction (ScRIA2 and ScRA) achieved the largest new bone volume and a 50 % bone marrow fraction (ScARA) resulted in new bone volume that was two times lower. In all the experimental groups, compression testing revealed the scaffolds to have a typical initial linear elastic range followed by a plastic region. Supplementary Video 7 shows representative biomechanical compression testing of the *in vivo* samples. Deformation of the scaffolds looked similar for the control and the *in vivo* scaffold and scaffold-BG combinations (Fig. 6B). Young's modulus was the largest in the Sc group with 50.02 ± 2.83 MPa and the smallest in the non-implanted control scaffolds with 46.12 ± 7.84 MPa and the ScARA group with 45.91 ± 8.92 MPa. The second highest Young's modulus was seen for scaffolds loaded with bone chips (ScRA), with 49.07 ± 2.20 MPa followed by BG harvested with the RIA 2 system (ScRIA2 group), with 47.36 ± 9.07 MPa. Notably, however, from the ANOVA analysis, no significant difference between the experimental groups was observed (*p* = 0.74), with particularly no significant difference between the ScRIA2 and the ScRA groups (*p* = 0.99). Note that the largest SD was observed in the ScRIA2 and ScARA groups (Fig. 6C). The largest yield strengths were found in the ScRIA2 (3.09 ± 0.51 MPa) and the ScRA (3.06 ± 0.17 MPa, *p* = 1) groups. These groups were followed by the non-implanted control scaffolds (3.02 ± 0.16 MPa) and the ScARA group (mean 2.83 ± 0.56 MPa) as well as the Sc group (mean 2.75 ± 0.48 MPa) (Fig. 6D). Again, no significant overall difference between the groups was observed in the ANOVA (*p* = 0.85), which is consistent with the hypothesis.

## 4 Discussion

The market for intramedullary BG harvesting technologies is evolving rapidly. In particular, there is an interest in strategies that allow separate bone marrow harvesting and thus the possibility of controlled transplantation in combination with bone chips [21]. This is the rationale behind advancing research into these strategies of intramedullary BG harvesting as an integral part of evolving the SGBR concept [1,5,7,36,39]. It is noteworthy that many attempts to induce or enhance bone formation with patient-derived cells, growth factors, and cytokines alone or in combination with 3D-printed implants have failed clinically [9]. It has been proposed that this may be due to the inability of endogenous and exogenous cells to remain alive and functional until the host vasculature has developed sufficiently to supply oxygen and nutrients and to remove metabolic waste [40]. Therefore, to test the novel ARA intramedullary BG harvesting concept, we have adhered to the clinically applied principle of SGBR using scaffolds in combination with freshly harvested BG – a combination that has impressive osteogenic potential, as well as advantages in terms of cost, regulations, and commercialization [1].

To investigate the successful translation of scaffold-BG construct concepts (SGBR), novel scaffolds such as the Voronoi design used in this study need to be sufficiently characterized to evaluate BG osteogenic capacity within context. Physical design is an important feature for the fabrication process of a scaffold for the facilitation of deposition of physiological tissue matrix that fosters bone regeneration. Particularly, the structure of the scaffold pores has a critical role in blood vessel growth and infiltration, cell growth, nutrient flow, and bone formation [41,42]. Pores with sizes >300–400 μm were found to enhance osteogenesis, vascularization, and oxygenation [42,43]. Accordingly, the pore structures of the mPCL-HA Voronoi scaffold as observed in this study were suitable to facilitate bone regeneration. Furthermore, appropriate filament integrity, no distinct HA particles, and a decentralized and uniformly distributed submicron HA particle rim were observed at the edges of the struts within the mPCL-HA in the μCT imaging. These are indicators that the applied gamma irradiation had no negative effect on scaffold morphology, which was further confirmed by SEM showing strut surfaces with appropriate integrity. Moreover, the mPCL-HA Voronoi scaffolds tested in this study exhibited a higher modulus of elasticity than the mPCL composite scaffolds currently used clinically for the treatment of long bone defects and 3D-printed with a rectilinear filling pattern [2,3] (Young's modulus of 22.2 MPa [16]). Stress–strain curves in the present study were observed to have very similar deformation behaviour to the PCL-tricalcium phosphate (wt 80 %:20 %) scaffolds with rectilinear layer structure [44], which have already been successfully used in clinical practice [2,3,45,46]. Complemented by an observed porosity of >50 % of the mPCL-HA Voronoi scaffolds, which we know to be fundamentally capable of facilitating the necessary neovascularization and new (bone) tissue formation [2,3,6], we have assessed the scaffolds as suitable for investigating and comparing different scaffold-BG constructs.

As we further develop the concept of SGBR with the novel ARA intramedullary harvesting concept and compare it with the RIA 2 system harvested BGs, it is important to investigate the interaction of BG and scaffold and therefore also the integration of scaffolds into the surrounding tissue in combination with the analysis of vascularization, fibrous encapsulation, or both [36]. Insertion of “foreign material” of scaffold-BG combination into the body causes the host tissue to initiate an inflammatory reaction known as “foreign body reaction” (FBR), characterized by a range of humoral and cellular activities [47]. Excessive FBR can lead to increased inflammation and extensive ECM synthesis, resulting in the formation of dense fibrotic tissue surrounding the implant, which prevents proper integration of the scaffold-BG constructs and host tissue, eventually leading to implant failure associated with failed bone defect treatment, further requiring additional (revision) surgery [47,48]. Here, no pronounced inflammatory elements or pathological signs of scaffold-BG construct encapsulation were observed macroscopically in any of the *in vivo* groups, with good tissue infiltration and neovascularization occurring in all the samples. Thus, it can be concluded that in addition to gamma irradiation being a suitable sterilization method for the scaffolds – as observed with other clinically used mPCL composite scaffolds [2,3,45] – successful integration into the surrounding tissue was achieved with the mPCL-HA Voronoi scaffold-BG constructs.

Furthermore, consistent with the literature [5,12], we observed that xenografting of fresh BG in the EBF model results in high viability and especially that osteoblasts and osteocytes survive and contribute to the formation of new (bone) tissue. Hence, these results confirm our findings from the preliminary study [5] and align with the findings of Corre et al. [12]. Corre et al. [12] observed in a nude mouse EBF model that biphasic calcium phosphate granules loaded with fresh BG from a transgenic rat strain resulted in the formation of abundant woven bone over implant surfaces [12]. At the cellular level, bones contain osteocytes buried in the mineralized matrix that live in microcavities (lacunae) and are interconnected by nanometre-sized channels called canaliculi [49]. This LCN of osteocytes enables cell-to-cell communication and transmits local mechanical stimuli, which in turn control bone formation and bone homeostasis [50]. Moreover, a comprehensive study (*in vitro* and *in vivo*) showed that osteocytes but not osteoblasts primarily deposit minerals around cell bodies and dendrites throughout the bone matrix (including the bone surface), in contrast to the widely held assumption that minerals are largely supplied externally by osteoblasts [51]. Notably, as described in previous studies [5,52,53], partially embedded osteocytes, also called osteoblast-osteocyte-like cells, were observed in the ECM next to BG fragments actively contributing to bone regeneration by recruiting osteoclast cells into areas of bone remodelling. These important observations demonstrate the osteoconductive and osteogenic potential of the mPCL-HA Voronoi scaffold-BG combinations to provide a favourable environment for the formation of stable hematomas and fibrin networks [54], cell adhesion, migration, proliferation and differentiation, and ultimately (bone) tissue ingrowth [4,55,56].

The ultrastructural composition of the ECM, which is a fundamental component of scaffold-BG constructs, is often neglected by research; however, it is as important as the molecular and cell biological aspects of SGBR [57]. In this study, we observed findings of multidirectional and lamellar patterns in the ECM of woven bone and bone chips, respectively, consistent with previous observations showing that in an organized ECM, such as on the lamellar surface of bone chips, the LCN is a highly organized structure and follows a similar orientation to the collagen fibres [58,59]. Conversely, in a disorganized ECM, such as woven bone between bone chips and in scaffold strut interstices, the LCN has a more chaotic arrangement [[60], [61], [62]]. Similar observations of mainly woven bone in areas of newly forming bone, such as around scaffold struts but more lamellar bone structure at the edges of solid bone structures (such as callus or bone chips), have been made in previous studies and confirm typical bone regeneration [63,64] with, notably, no qualitative differences between the investigated scaffold-BG groups. Further, CLSM showed that the tissue surrounding the mineralized bone chips was intensely stained with rhodamine, in contrast to the bone chips themselves, where only the osteocyte LCN was stained. Thus, the complex microenvironment of fresh BG, consisting of osteoconductive and osteoinductive bone matrix and osteogenic capacity of the cellular components, was shown to be responsible for successful bone regeneration.

The surviving BG-residing osteocytes benefit most from the microenvironment and actively participate in bone regeneration. While ultrastructural features of mature bone were not detectable in more distant areas, overall, our results indicate new formation of physiological bone on the bone chips in all the scaffold-BG construct groups. It may be noted that the groups with a higher bone marrow content (the ScRIA2 and ScARA groups) show more variability in total bone volume and new bone volume. Similar to our study, previous studies also showed that while the bone marrow is very complex with many valuable osteoinductive proteins [21], it has already been shown that greater osteogenicity emanates from bone chip cells [65]. Thus, the use of bone marrow has been shown to be an effective surgical indication for bridging small bony gaps, such as in spinal fusion [66,67] and healing of tibial fractures [68,69]. However, its rather small total cell pool [70] and limited capacity for controlled release of osteogenic proteins [21] provide clues as to why we also observed greater variability in bone regeneration in the ScARA group compared to the ScRIA2 and ScRA groups in this study. This also demonstrates why BGs with high bone chip content rather than bone marrow are used in the current clinical cases of SGBR [2,3,7,39] and why further development of concepts of intramedullary harvesting of endosteal bone chips (such as the ARA intramedullary harvesting concept [21]) are highly relevant.

We note some of the limitations of this study. In histological samples, bone tissue formation was not quantitatively assessed. The use of specific markers for sheep and rats for IHC would allow the extent of new bone formation and differentiation of origin (i.e., sheep versus rat) to be determined and may therefore be the subject of future studies as this was not done in this study. Moreover, the fluorochromes used for bone labelling are calcium-chelating substances that insert into the mineralizing front of mineralizing surfaces, and they can be used to confirm active bone regeneration, and one label is sufficient for this purpose [33]. Hence, the study with fluorescent xylenol orange could confirm an active bone regeneration and thus the viability of the BG and osteogenic microenvironment demonstrated in the SEM analysis. However, the quantification of new bone formation with a single fluorochrome dye is not useful because in histological sections, the width of a fluorochrome band also depends on the angle at which a 3D labelling plane is sectioned. Hence, serial fluorochrome dye injection needs to be included in future studies with orthotopic mPCL-HA Voronoi scaffold-BG construct implantation. Furthermore, orthotopic defect models in rats indeed allow the testing of BGs in femoral defects [71]. The principal apparent advantage is testing in the most translatable anatomic site, particularly because of the biomechanical stimuli associated with orthotopic implantation of scaffold-BG constructs that cannot be reproduced in an ectopic implantation site. However, xenograft implantation of scaffold-BG constructs in rats would still have the limitations associated with the use of xenografts. Therefore, future studies, which have already been initiated by our research group, aim for further testing of (orthotopically implanted) BG-scaffold (Generation 4.0 scaffold) constructs using the novel ARA harvesting concept in a world-leading sheep segmental defect model >12 months study period [35].

## 5 Conclusion

Building upon our promising preliminary work [5,30], we have shown in this study that the design of mPCL-HA Voronoi scaffolds in combination with standard RIA 2 system BG as well as alternatively intramedullary harvested BGs is optimized for bone regeneration. It is designed and fabricated based on the recognized key properties of a suitable macroporous structure and osteoconductive properties to advance scaffold-BG construct-based SGBR. A synergistic enhancement of the bone regeneration potential of mPCL-HA Voronoi scaffolds in combination with BG was observed, with BGs harvested with the novel ARA harvesting concept (the ScRA and ScARA groups) being comparable to the gold standard BG harvesting of the RIA 2 system (the ScRIA2 group). Importantly, BG harvested with the novel intramedullary ARA harvesting concept and used in mPCL-HA Voronoi scaffold-BG constructs demonstrated comparable biocompatibility, bone regeneration capacity, and biomechanical properties to BG from the RIA 2 system.

## Funding sources

Financial support for this project was provided by the Australian Research Council (10.13039/501100000923ARC) via the 10.13039/501100000923ARC Training Centre for Multiscale 3D Imaging, Modelling and Manufacturing (M3D Innovation, project 10.13039/100011546IC 180,100,008) and the Max Planck Queensland Centre (MPQC) for the Materials Science of Extracellular Matrices.

## Declaration of competing interest

DWH is a cofounder of BellaSeno GmbH, an ISO 13485-certified medical technology company and a cofounder and shareholder of Osteopore International. The remaining authors declare that the research was conducted in the absence of any commercial or financial relationships that could be construed as potential conflicts of interest.

## Data availability

Data will be made available upon reasonable request.

## CRediT authorship contribution statement

**Markus Laubach:** The last two authors contributed equally to this work and share senior authorship. **Buddhi Herath:** Conceptualization, Methodology, Formal analysis, Investigation, Data curation, Visualization, Writing – original draft. **Sinduja Suresh:** Methodology, Investigation, Writing – review & editing. **Siamak Saifzadeh:** Investigation, Writing – review & editing. **Bronwin L. Dargaville:** Conceptualization, Funding acquisition, Project administration, Writing – review & editing. **Silvia Cometta:** Conceptualization, Methodology, Writing – review & editing. **Victoria Schemenz:** Investigation, Writing – review & editing. **Marie-Luise Wille:** Investigation, Writing – review & editing. **Jacqui McGovern:** Investigation, Funding acquisition, Formal analysis, Writing – review & editing. **Dietmar W. Hutmacher:** Investigation, Formal analysis, Writing – review & editing. **Flavia Medeiros Savi:** Conceptualization, Resources, Methodology, Investigation, Supervision, Funding acquisition, Project administration, Writing – review & editing. **Nathalie Bock:** Conceptualization, Formal analysis, Visualization, Supervision, Project administration, Writing – review & editing.
