# Supplementary material for: An innovative intramedullary bone graft harvesting concept as a fundamental component of scaffold-guided bone regeneration: A preclinical in vivo validation
Source: J Orthop Translat. 2024 Jun 5;47:1–14. doi: 10.1016/j.jot.2024.05.002 (PMC11215842; doi:10.1016/j.jot.2024.05.002)
Supplement: Multimedia component 1 [file mmc1.pdf]

Supplement

Contents

Supplementary tables .... - 2 -

Supplementary Table 1. Primary antibodies and protocol specifications for immunohistochemistry markers used in this study. .... - 2 -

Supplementary Table 2. Detailed explanations and descriptions on the morphological assessment of mineralized samples, including the evaluation of active bone regeneration. - 3 -

Supplementary Table 3. Overview of the number of samples of the respective experimental group that were used for the different analysis methods. µCT, micro-computed tomography. .... - 5 -

Supplementary figures .... - 6 -

Supplementary Figure 1. SEM images depicting the scaffold Generations 1.0 - 4.0 designed and manufactured for the concept of scaffold-guided bone regeneration. .... - 6 -

Supplementary Figure 2. Stereolithography (STL) file of the Voronoi scaffold design. ... - 7 -

Supplementary Figure 3. Presentation of scaffold porosity calculation. .... - 8 -

Supplementary Figure 4. Representative images of the workflow in ImageJ software to calculate the pore size and strut diameter of the µCT data of the pristine scaffolds. ...... - 8 -

Supplementary Figure 5. Setup for assessment of the (bio)mechanical characteristics of the pristine mPCL-HA scaffolds as well as the *ex vivo* samples. .... - 9 -

Supplementary Figure 6. Assessment of morphology of 3D-printed mPCL-HA Voronoi scaffolds. .... - 10 -

Supplementary Figure 7. Surface characterization of mPCL-HA Voronoi scaffolds. .... - 11 -

Supplementary Figure 8. Representative histological sections of the new forming tissue (ScRA group) stained with H&E, COL I and OC, depicting overlapping and interrelating phases of bone remodeling. .... - 12 -

Supplementary Figure 9. Representative confocal laser microscopy of rhodamine and second harmonic generation analysis. Origin of the images according to the experimental groups: A-B, ScARA group; C-D, ScRA group; E-F, ScRIA2 group. .... - 13 -

Supplementary Figure 10. SEM imaging of the bone chips showed a viable osteocyte network within the graft fragments. .... - 14 -

References .... - 16 -

Supplementary tables

Supplementary Table 1. Primary antibodies and protocol specifications for immunohistochemistry markers used in this study.

Antibody	Specimen tissue site	Reactivity	Raised in	Clonality	Cat. no.	Antigen retrieval	Blocking	Dilution	In-cubation	DAB	Description
CD68 (Cluster of Differentiation 68)	Rat	Mouse, rat	Rabbit	Polyclonal	ab125212	Proteinase K 5 min	2% BSA 30 min	1:300	1hr	0:25 min	M1 and M2 macrophage marker
iNOS (nitric oxide synthase)	Rat	Mouse, rat	Rabbit	Polyclonal	ab15323	Proteinase K 5 min	2% BSA 30 min	1:100	1hr	2:45 min	M1 macro-phage, pro-inflammatory
MR (mannose receptor)	Rat	Mouse, rat, human	Rabbit Mouse	Polyclonal	ab64693	Proteinase K 5 min	2% BSA 30 min	1:100	1hr	0:40 min	M2 macro-phage, pro-regenerative
vWF (von Willebrand factor)	Rat	Human	Rabbit	Polyclonal	IR527	Proteinase K 5 min	2% BSA 30min	Ready to use	1hr	2:30 min	Mature Blood vessel
COL I (collagen type I)	Rat	Mouse, rat, sheep, goat, horse, cow, human, pig	Rabbit	Monoclonal	ab138492	Proteinase K 5 min	2% BSA 30 min	1:100	1hr	0:20 min	Early bone marker for osteoblastic differentiation
COL II (collagen type II)	Rat	Human, mouse, sheep	Mouse	Monoclonal	DSHB II-II6B3	Proteinase K 5 min	2% BSA 30 min	1:100	1hr	1:00 min	Endochondral bone formation at areas of cartilage matrix
OC (osteocalcin)	Rat	Human	Rabbit	Monoclonal	ab 133612	Proteinase K 5 min	2% BSA 30 min	1:100	1hr	0:30 min	Late osteogenic bone marker

Supplementary Table 2. Detailed explanations and descriptions on the morphological assessment of mineralized samples, including the evaluation of active bone regeneration.

Focus	Assessment technique	Detailed explanation and description of the procedure
**Assessment of active bone regeneration**	(1) SEM with resin cast etching to visualize the surface morphological features of the BGs and osteocyte network	Scanning electron microscopy provides very high spatial resolution and a wide field of view, allowing the characterization of bone microstructure. *In vivo* performance of biomedical implant materials can be assessed in detail using SEM [1, 2] and particularly the evaluation of the osteocyte network within implanted biomaterials [3, 4, 5]. Moreover, osteocyte LCN can be well depicted on resin-cast etched ground sections [5, 6] through osteocyte etching (removal of the inorganic phase with phosphoric acid (H_3PO_4) and digestion of the organic phase with sodium hypochlorite (NaOCl)).
	(2) Confocal fluorescence imaging of xylenol orange to qualitatively confirm bone regeneration	Resin blocks of fluorochrome-labelled tissue were protected from intense light during processing, stored in the dark, and 50 µm thick ground sections were used for further analysis. The 50 µm ground resin sections stained with xylenol orange were imaged at the maximum intensity projections of the z-stacks. Images were acquired at 2.5 µm intervals along the z-axis on a Nikon TiE inverted widefield microscope (Nikon, Tokyo, Japan) through a Nikon Plan Apo 10x, 0.45 NA DIC L objective. The fluorescence filters used were a Nikon FITC filter set (excitation 465–495 nm, 505 nm dichroic beam splitter, emission 515–555 nm) and a Nikon TRITC filter set (excitation 528–552 nm, 565 nm dichroic beam splitter, emission 558–632 nm). Exposure times were 2 ms for the FITC channel and 30 ms for the FITC channel.
**In-depth assessment collagen orientation**	(3) Histological analysis using modified Goldner's trichrome (GT) staining	Modified GT staining offers the distinct benefit of overall tissue morphology due to sharply distinguishing mature bone matrix, immature new bone matrix, and calcified cartilage [7]. Standard laboratory protocols [8, 9] were applied.
	(4) Rhodamine staining for in-depth analysis of osteocytes lacuno-canalicular network (LCN)	The rhodamine molecule is small enough (~0.9 nm) to penetrate the cortical bone via the canaliculi [10] and binds to osseous surfaces. Sample preparation, staining procedure, and imaging techniques have been described in detail elsewhere [11]. Resin blocks were used to visualize osteocytes and the LCN with rhodamine staining through CLSM. Briefly, the samples were incubated in 70% ethanol for 5 days, 80%

	(5) Confocal laser scanning microscopy (CLSM) for orientation of collagen fibres was assessed using second-harmonic generation (SHG) signal	ethanol for 5 days, and 90% ethanol for 9 days. Next, rhodamine 6G (#R4127-5G, Sigma) was diluted in 100% ethanol (250 mL 100% ethanol mixed with 1.25 g of rhodamine 6G for 30 min) and the samples were incubated protected from light for 3 days. The samples were then cleared in 2 changes of xylene for 4 h each and incubated in resin pre-infiltration solution for 7 days, followed by incubation in infiltration solution for 7 days (for details of solution, see [8]). The samples were resin embedded under vacuum and left to polymerize at 4 °C for 7 days. Briefly, the rhodamine-stained and polymethyl methacrylate (PMMA) resin-embedded samples were imaged with fluorescence CLSM (Leica TCS SP8 DLS Multiphoton, Wetzlar, Germany), equipped with an oil immersion lens (Leica, HCX PL APO 40× NA 1.25 oil) using argon laser light ($\lambda_{excitation}$ = 514 nm / $\lambda_{emission}$ = 550–650 nm). The confocal microscope was equipped with a Mai Tai HP multiphoton laser, which was used to visualize the SHG signal of fibrillar collagen of the same resin blocks. Second-harmonic generation excitation wavelength was set at 910 nm and detection wavelength at 450–460 nm. A strong SHG signal is evident in regions with dense and well-aligned collagen fibrils perpendicular to the incoming light.

Supplementary Table 3. Overview of the number of samples of the respective experimental group that were used for the different analysis methods. µCT, micro-computed tomography.

**Analysis (number of samples)**	**Sc**<br>Scaffold alone	**ScRIA2**<br>Scaffold with RIA 2 system bone graft	**ScRA**<br>Scaffold with RA option bone graft	**ScARA**<br>Scaffold with ARA option bone graft
µCT imaging	8	8	7	9
Histological analysis	1	2	2	2
Biomechanical testing	7	6	5	8

Supplementary figures

Supplementary Figure 1. SEM images depicting the scaffold Generations 1.0 - 4.0 designed and manufactured for the concept of scaffold-guided bone regeneration.

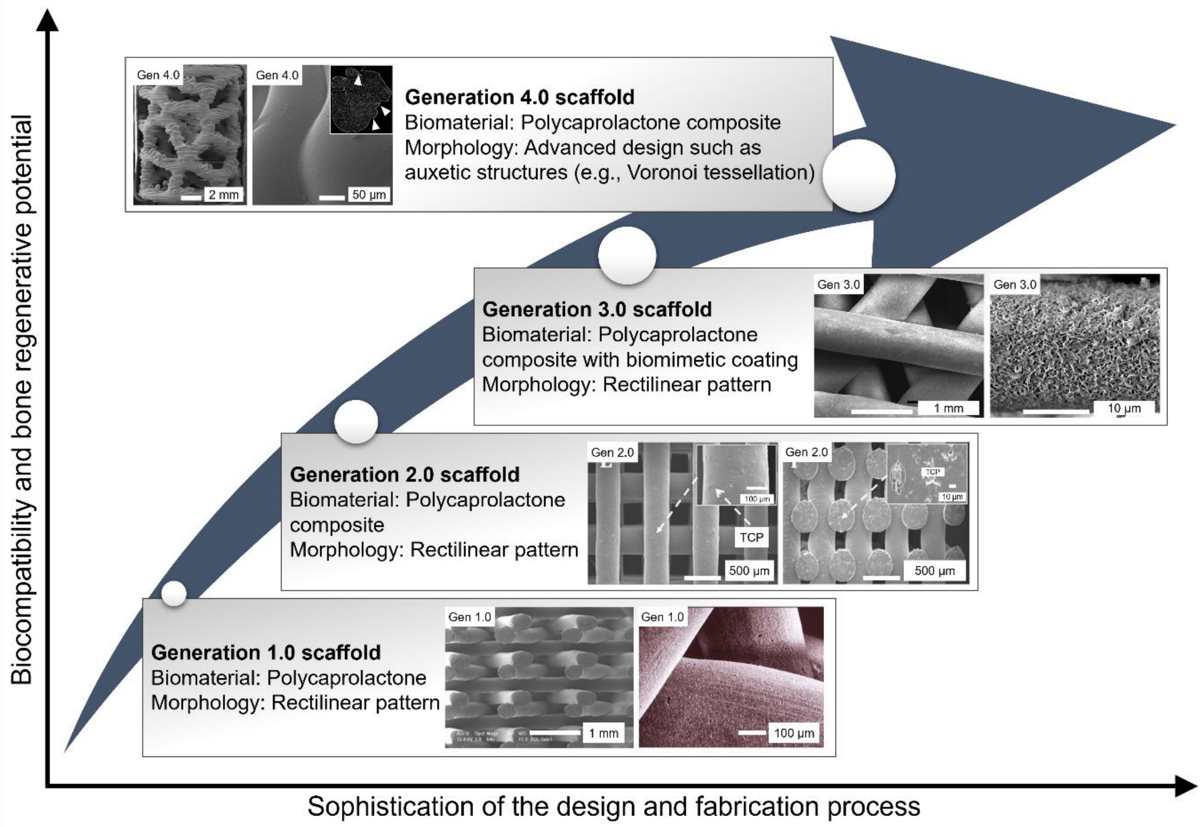

For detailed explanations of the various scaffold generations, we refer to the supplement of Ref [12]. Image reprinted from supplement of Ref [12].

Supplementary Figure 2. Stereolithography (STL) file of the Voronoi scaffold design.

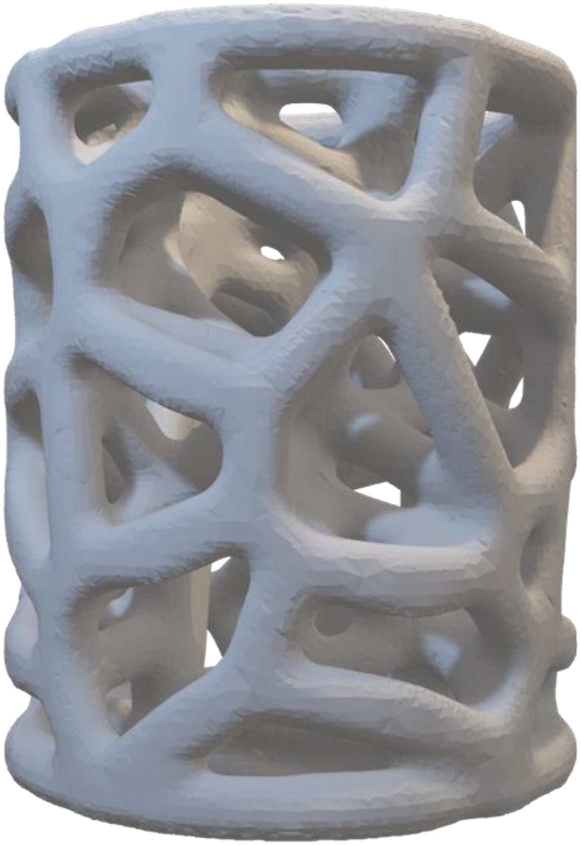

Supplementary Figure 3. Presentation of scaffold porosity calculation.

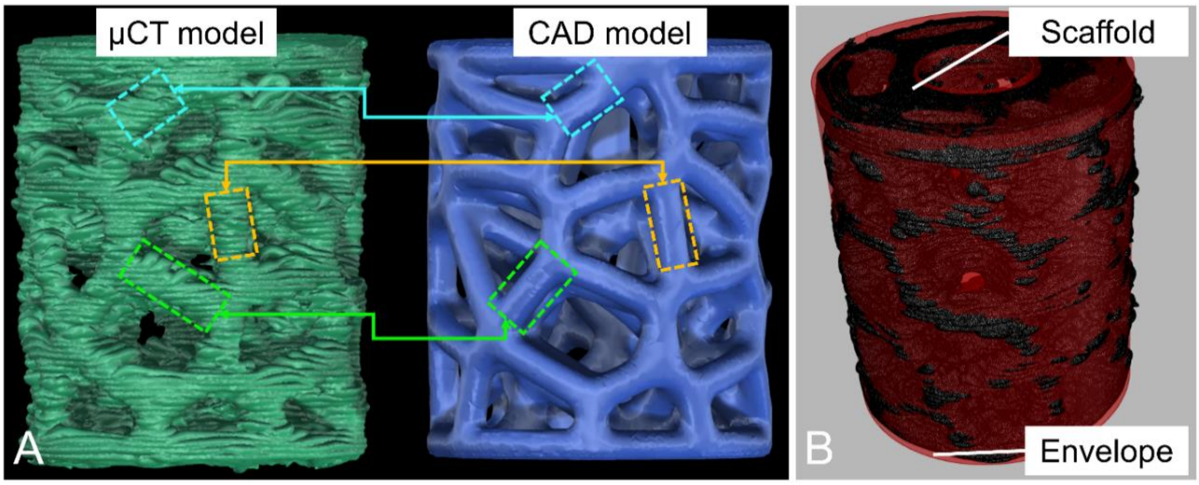

Due to thicker struts in the µCT model, a mean porosity (P) of 50.44% (SD 3.53) is observed compared to the computer aided design (CAD) model porosity of 57.79% (A). Visualization of the calculation of the porosity of the µCT model: an envelope is sculpted around the scaffold and then the scaffold volume is subtracted from the volume that fills the envelope (B). The porosity of a porous scaffold structure refers to the percentage of its internal pore volume to its total volume applying the following porosity calculation equation:

$$P\ (\%) = \frac{VE - VS}{VE} \times 100$$

Among them, P is the porosity (%) of the porous structure, VE is the total volume of the envelope, and VS is the volume of the scaffold.

Supplementary Figure 4. Representative images of the workflow in ImageJ software to calculate the pore size and strut diameter of the µCT data of the pristine scaffolds.

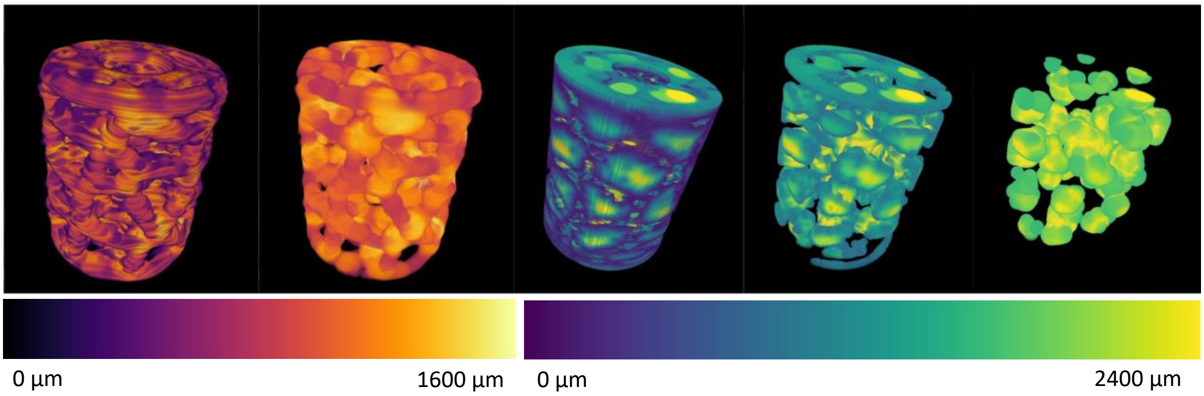

Given the irregularity of the filaments deposited during 3D printing of the scaffolds, to gauge the strut and pore diameters, the local thickness map of the samples was calculated. The local thickness map calculates the maximum inscribed sphere that can be located inside the given model. In the case of the scaffold, this measures the diameters of the struts as well as the nodes. The µCT images were imported into the open-source image processing program ImageJ (National Institute of Health, USA) where the calculation was performed for both the scaffold and the pore regions. This figure displays the local thickness maps for the scaffold struts and the pores with varied diameter thresholds.

Supplementary Figure 5. Setup for assessment of the (bio)mechanical characteristics of the pristine mPCL-HA scaffolds as well as the *ex vivo* samples.

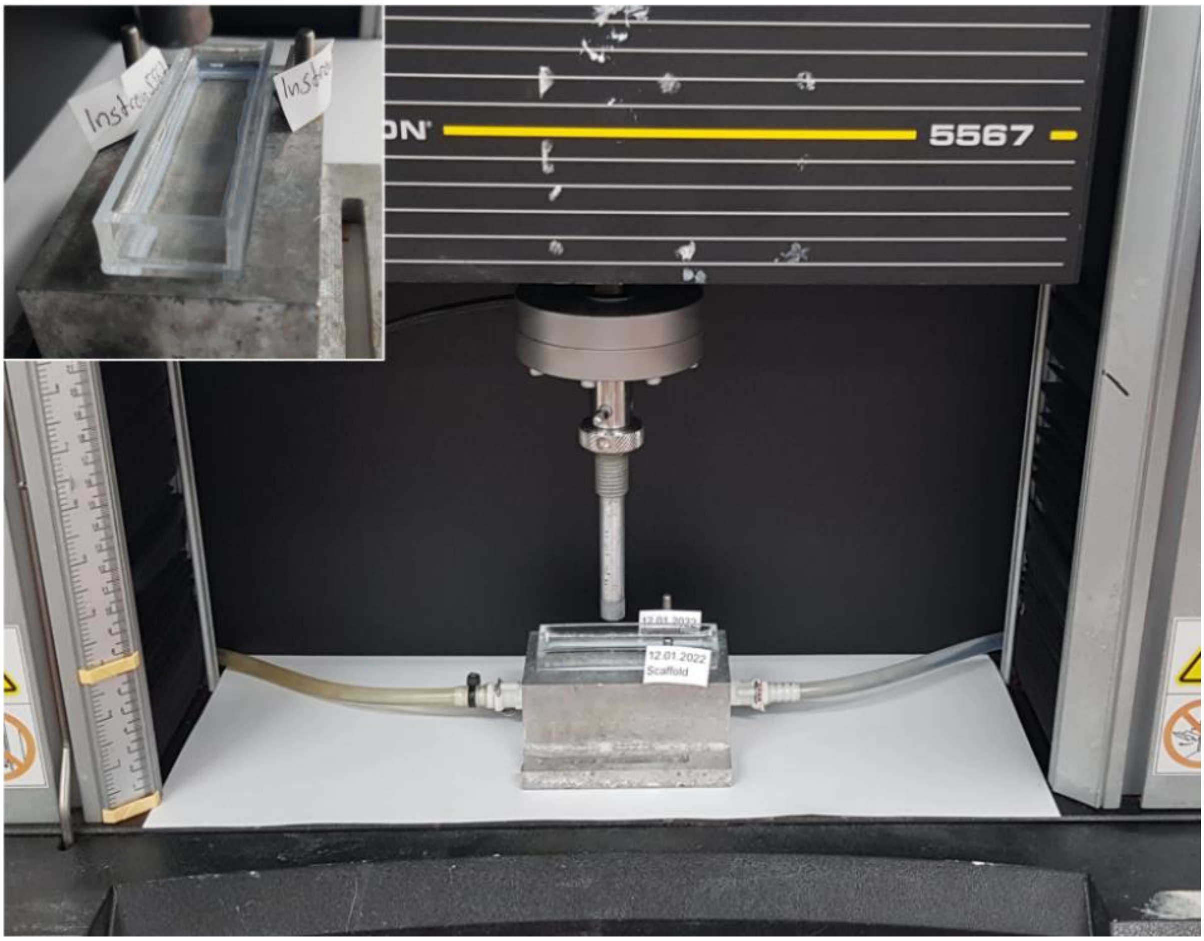

Mechanical compression testing conducted under simulated physiological conditions with constant water temperature of 37°C using a 1X PBS bath (pH 7.4). PBS, phosphate buffered saline.

Supplementary Figure 6. Assessment of morphology of 3D-printed mPCL-HA Voronoi scaffolds.

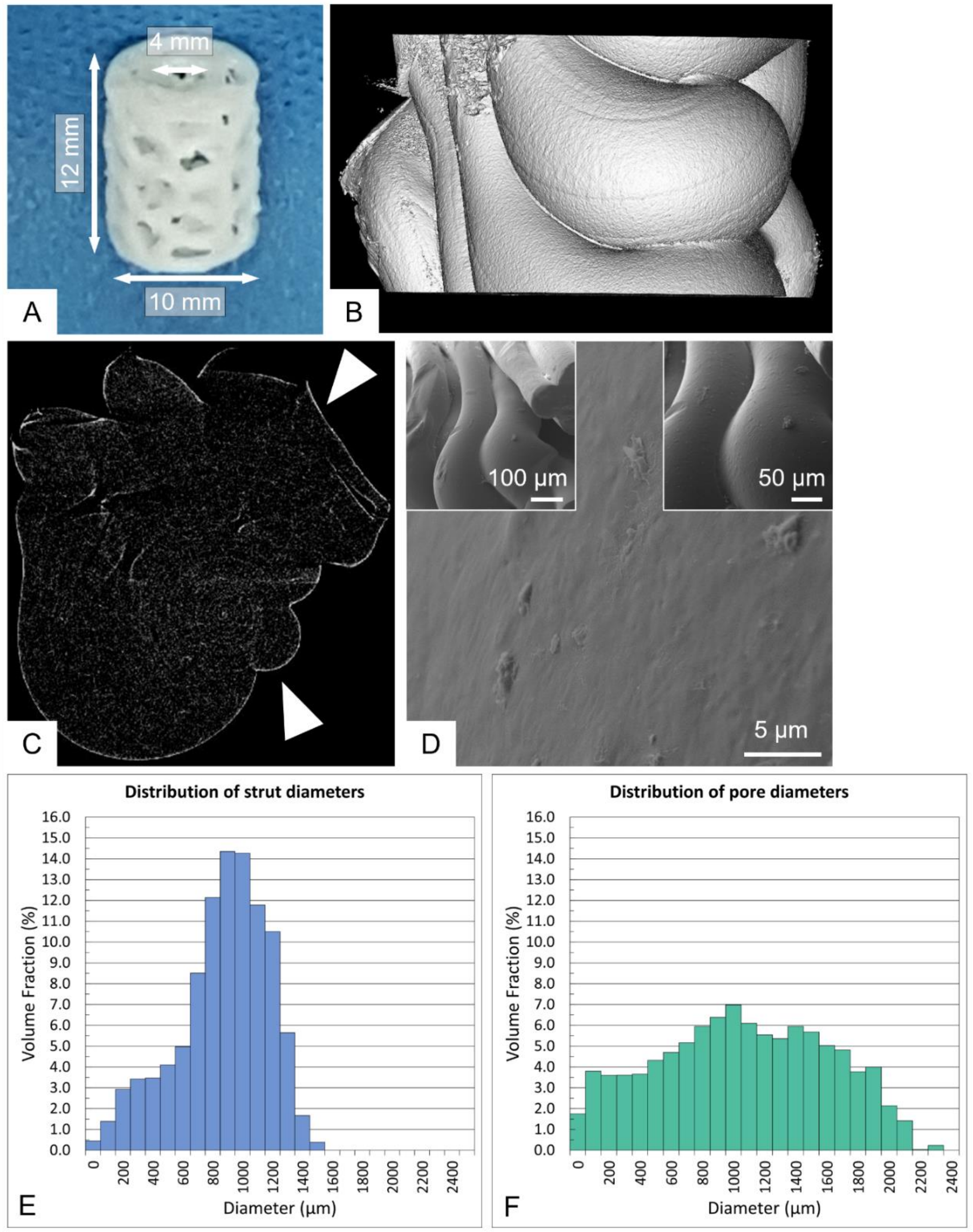

Macroscopic evaluation shows high printing accuracy with fully interconnected highly porous strut network (A) with smooth surface and adequate layering pattern confirmed in segmented µCT data (B). HA particles were predominantly at the filament edges (white arrows) (C) and SEM imaging shows appropriate filament and scaffold surface properties (D). Strut (E) and pore (F) diameter of mPCL-HA Voronoi scaffolds showed normal distribution of data (n = 6 samples). The histograms of the local thickness display both the strut diameters (E) and the pore diameters (F) against their respective volume fractions. The averaged mean of the strut diameters and the pore diameters were 1.10 mm and 0.92 mm respectively. The majority of the struts seem to have a diameter of 0.70 mm – 1.25 mm but the pore diameters show a distribution that is evenly spread between 0.05 mm – 2.10 mm.

Supplementary Figure 7. Surface characterization of mPCL-HA Voronoi scaffolds.

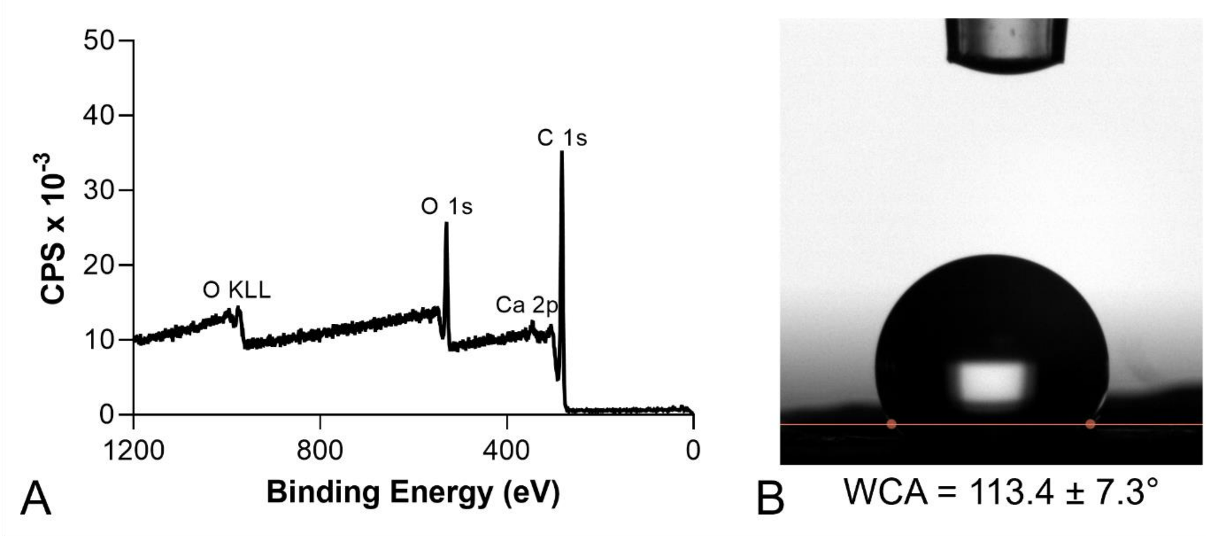

Exemplary plot of XPS wide spectra evidencing the presence of elemental oxygen, carbon and calcium (A). Surface wettability of mPCL-HA scaffolds (n = 6) showing a WCA of 113.4 ± 7.3° (B). WCA, water contact angle; XPS, X-ray Photoelectron Spectroscopy.

Supplementary Figure 8. Representative histological sections of the new forming tissue (ScRA group) stained with H&E, COL I and OC, depicting overlapping and interrelating phases of bone remodeling.

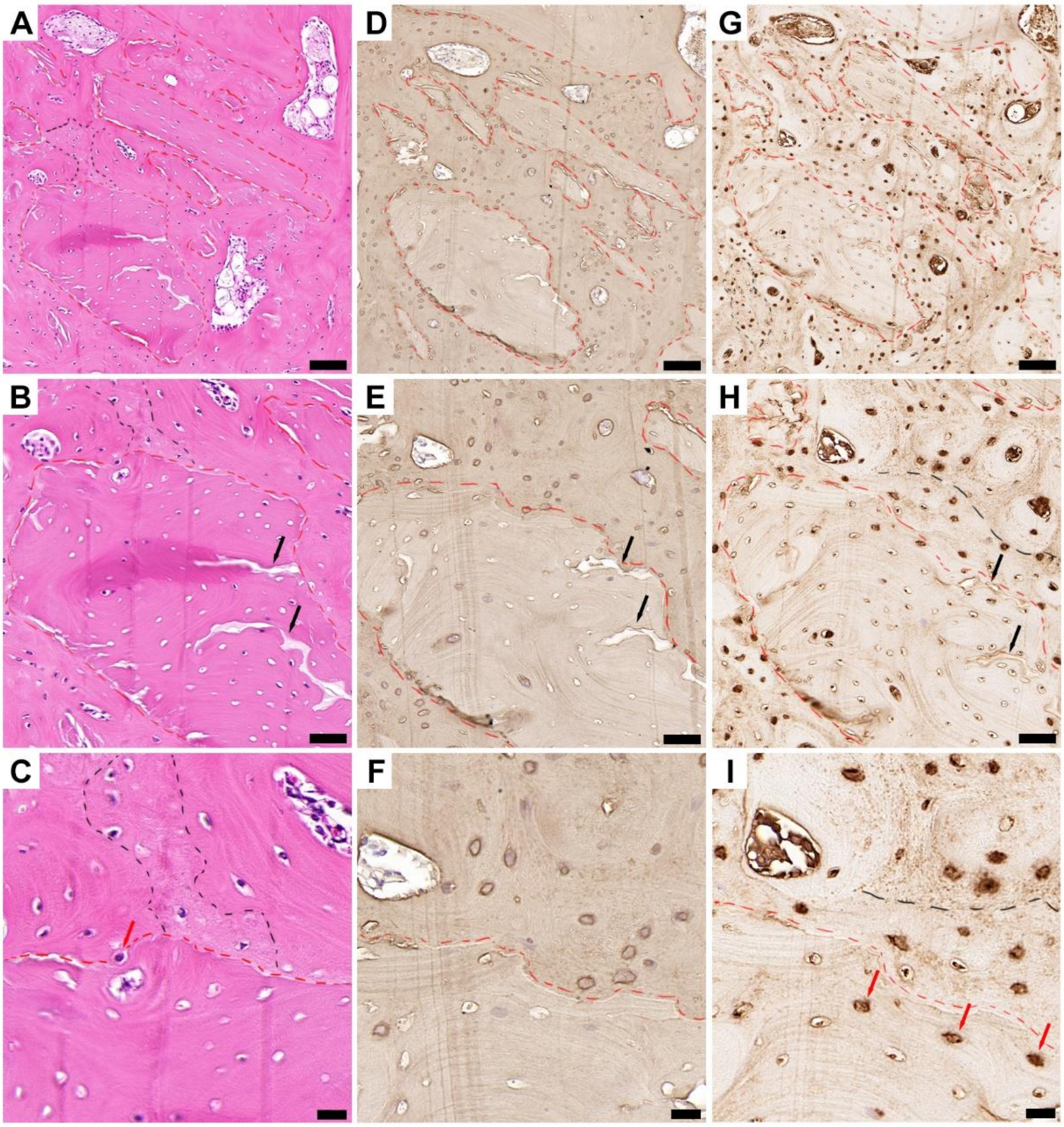

Sections of the new forming tissue stained with H&E (A-C), COL I (D-F) and OC (G-I), depicting overlapping phases of bone remodeling around the grafted material, showing fragments of the graft material (red dashed lines), osteochondral bone formation (black arrows) as well as woven bone (black dashed line). The graft material appears well incorporated within the tissue, alive and viable as demonstrated by OC staining at osteoblast cells (I, red arrows). Yet, the intervening space between bone graft fragments was bridged with newly forming bone. COL I, collagen type I; OC, osteocalcin. Scale bars: A, D, E, 100 µm; B, E, H, 50 µm; C, F; I, 20 µm.

Supplementary Figure 9. Representative confocal laser microscopy of rhodamine and second harmonic generation analysis. Origin of the images according to the experimental groups: A-B, ScARA group; C-D, ScRA group; E-F, ScRIA2 group.

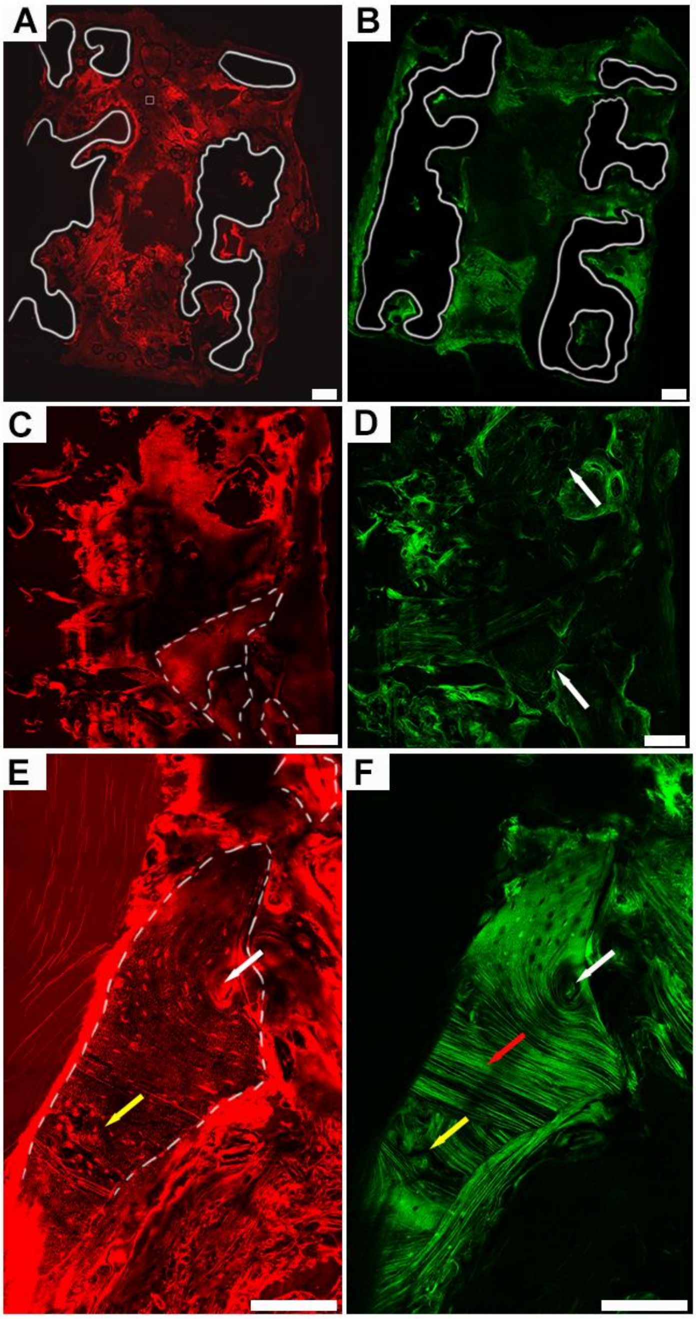

Overview images of the newly formed tissue, showing collagen the ultrastructural morphology of the bone fragments and collagen fibers orientation (A, B). Fragments of the graft material are observed within the tissue (C, dashed line), as well as collagen fibers orientation (D, white arrow). Higher magnification of the characteristic structure of lamellar bone (E, white dashed line), with a partially grafted osteon (E, white arrow) and its radially organized LCN next to the lamellar structured tissue. In the same section, an area of woven bone can also be identified next to the lamellar structure (E, yellow arrow). The collagen fibers within the same areas appeared to be very organized and thick (F, green signal), however, where bone remodeling is taking place, the collagen fibers appear to be thin and randomly organized (F, white arrow). Scale bars: A, B, 1 mm; C, D, 250 µm; E, F, 200 µm.

Supplementary Figure 10. SEM imaging of the bone chips showed a viable osteocyte network within the graft fragments.

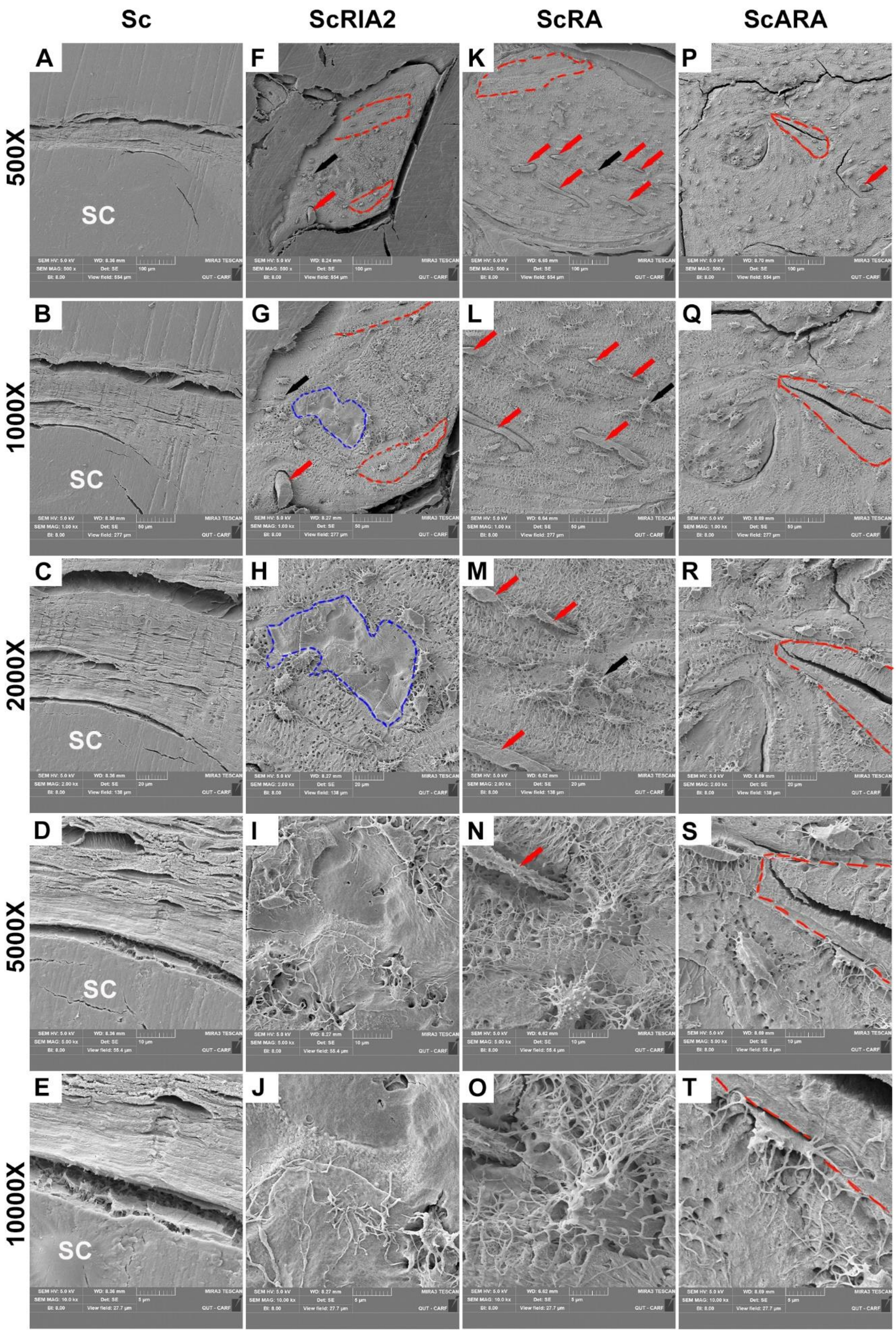

Ultrastructural morphology of the new tissue formed around the scaffold alone (Sc group), and variations in the architecture of new bone matrix of groups (groups: ScRIA2, ScRA and ScARA). SEM analysis of Sc group showed thick collagen fibers aligned on the outer surface of the scaffold strut (A-E). The remnants of the grafted material (F, G, K, P-T, red dashed line), the lamellar bone appears less cellular. Areas of woven bone presented several grouped osteoblastic-osteocyte like (F, G, K-M, black arrows). Some remnants of mineralized cartilage were also observed (G-H, blue dashed line), within a well vascularized tissue (red arrows).

References

1. Palmquist A. A multiscale analytical approach to evaluate osseointegration. J Mater Sci Mater Med 2018;29(5):60. [eng].
2. Shah FA, Thomsen P, Palmquist A. Osseointegration and current interpretations of the bone-implant interface. Acta Biomaterialia 2019;84:1-15.
3. Shah FA, Snis A, Matic A, Thomsen P, Palmquist A. 3D printed Ti6Al4V implant surface promotes bone maturation and retains a higher density of less aged osteocytes at the bone-implant interface. Acta Biomaterialia 2016;30:357-67.
4. Shah FA, Johansson ML, Omar O, Simonsson H, Palmquist A, Thomsen P. Laser-Modified Surface Enhances Osseointegration and Biomechanical Anchorage of Commercially Pure Titanium Implants for Bone-Anchored Hearing Systems. PLOS ONE 2016;11(6):e0157504.
5. Sparks DS, Savi FM, Dlaska CE, Saifzadeh S, Brierly G, Ren E, *et al.* Convergence of scaffold-guided bone regeneration principles and microvascular tissue transfer surgery. Sci Adv 2023;9(18):eadd6071. [eng].
6. Kubek DJ, Gattone Ii VH, Allen MR. Methodological assessment of acid-etching for visualizing the osteocyte lacunar-canalicular networks using scanning electron microscopy. Microscopy Research and Technique 2010;73(3):182-86.
7. Gruber HE. Adaptations of Goldner's Masson Trichrome Stain for the Study of Undecalcified Plastic Embedded Bone. Biotechnic & Histochemistry 1992;67(1):30-34.
8. Sparks DS, Saifzadeh S, Savi FM, Dlaska CE, Berner A, Henkel J, *et al.* A preclinical large-animal model for the assessment of critical-size load-bearing bone defect reconstruction. Nat Protoc 2020;15(3):877-924. [eng].
9. Henkel J, Medeiros Savi F, Berner A, Fountain S, Saifzadeh S, Steck R, *et al.* Scaffold-guided bone regeneration in large volume tibial segmental defects. Bone 2021;153:116163.
10. Fritton SP, Weinbaum S. Fluid and Solute Transport in Bone: Flow-Induced Mechanotransduction. Annual Review of Fluid Mechanics 2009;41(1):347-74.
11. Moreno-Jiménez I, Garske DS, Lahr CA, Hutmacher DW, Cipitria A. Targeted 2D histology and ultrastructural bone analysis based on 3D microCT anatomical locations. MethodsX 2021;8:101480.
12. Laubach M, Herath B, Bock N, Suresh S, Saifzadeh S, Dargaville BL, *et al. In vivo* characterization of 3D-printed polycaprolactone-hydroxyapatite scaffolds with Voronoi design to advance the concept of scaffold-guided bone regeneration. Frontiers in Bioengineering and Biotechnology 2023;11. [English].
